# *Vibrio neptunius* Produces Piscibactin and Amphibactin and Both Siderophores Contribute Significantly to Virulence for Clams

**DOI:** 10.3389/fcimb.2021.750567

**Published:** 2021-10-25

**Authors:** Fabián Galvis, Lucía Ageitos, Jaime Rodríguez, Carlos Jiménez, Juan L. Barja, Manuel L. Lemos, Miguel Balado

**Affiliations:** ^1^ Departamento de Microbiología y Parasitología, Instituto de Acuicultura y Facultad de Biología-CIBUS, Universidade de Santiago de Compostela, Santiago de Compostela, Spain; ^2^ Centro de Investigacións Científicas Avanzadas (CICA) e Departamento de Química, Facultad de Ciencias, Universidade da Coruña, A Coruña, Spain

**Keywords:** Coralliilyticus, *Vibrio neptunius*, bivalve molluscs pathogens, virulence factors, siderophores, piscibactin, amphibactin, aquaculture

## Abstract

*Vibrio neptunius* is an inhabitant of mollusc microbiota and an opportunistic pathogen causing disease outbreaks in marine bivalve mollusc species including oysters and clams. Virulence of mollusc pathogenic vibrios is mainly associated with the production of extracellular products. However, siderophore production is a common feature in pathogenic marine bacteria but its role in fitness and virulence of mollusc pathogens remains unknown. We previously found that *V. neptunius* produces amphibactin, one of the most abundant siderophores in marine microbes. In this work, synthesis of the siderophore piscibactin was identified as the second siderophore produced by *V. neptunius*. Single and double mutants in biosynthetic genes of each siderophore system, piscibactin and amphibactin, were constructed in *V. neptunius* and their role in growth ability and virulence was characterized. Although the High Pathogenicity Island encoding piscibactin is a major virulence factor in vibrios pathogenic for fish, the *V. neptunius* wild type did not cause mortality in turbot. The results showed that amphibactin contributes more than piscibactin to bacterial fitness *in vitro*. However, infection challenges showed that each siderophore system contributes equally to virulence for molluscs. The *V. neptunius* strain unable to produce any siderophore was severely impaired to cause vibriosis in clams. Although the inactivation of one of the two siderophore systems (either amphibactin or piscibactin) significantly reduced virulence compared to the wild type strain, the ability to produce both siderophores simultaneously maximised the degree of virulence. Evaluation of the gene expression pattern of each siderophore system showed that they are simultaneously expressed when *V. neptunius* is cultivated under low iron availability *in vitro* and *ex vivo*. Finally, the analysis of the distribution of siderophore systems in genomes of *Vibrio* spp. pathogenic for molluscs showed that the gene clusters encoding amphibactin and piscibactin are widespread in the Coralliilyticus clade. Thus, siderophore production would constitute a key virulence factor for bivalve molluscs pathogenic vibrios.

## Introduction

Bacteria belonging to the genus *Vibrio* (Vibrios) are ubiquitously distributed in the marine environment and are also a dominant fraction of bivalve microbiota (Vezzulli et al., 2018). Mollusc hemolymph is a critical site for the host immune response (Potgieter et al., 2015). Bivalve hemocytes can kill vibrios by phagocytosis and production of reactive oxygen species, highly reactive nitric oxide, antimicrobial peptides and hydrolytic enzymes (Destoumieux-Garzón et al., 2020). Interestingly, some Vibrios are part of the resident microbiota of bivalves as they persist in the hemolymph in the absence of an environmental source of population (Potgieter et al., 2015; Lokmer et al., 2016; Vezzulli et al., 2018; Zhang et al., 2018). The microbiota benefits the host as it boosts the immune system, and promotes reproduction, nutrition and defence mechanisms (Engel et al., 2002; McFall-Ngai et al., 2013; Cahill et al., 2016; Utermann et al., 2018). Nonetheless, under unfavourable conditions some bacteria are responsible for disease outbreaks. Among them, vibriosis is a serious epizootic disease caused by some *Vibrio* spp. that has become the most important limiting factor of the intensive fish and shellfish mariculture industry worldwide (Paillard et al., 2004; Toranzo et al., 2005; Travers et al., 2015; Dubert et al., 2017).

*Vibrio* species belonging to the Coralliilyticus and Orientalis clades are among the best-known species of bivalve pathogens (Dubert et al., 2017). They include *V. neptunius*, a marine bacterium that was isolated from marine water samples and animals such as turbot larvae (*Scophthalmus maximus*), rotifers (*Brachiomus plicatilis*) and larval stages of cephalopods (*Octopus vulgaris*) (Thompson et al., 2003; García-Amado et al., 2011; Farto et al., 2019). Notably, this bacterium is a relevant pathogen of aquacultured marine invertebrates, including artemia, oysters and clams (Prado et al., 2005; Kesarcodi-Watson et al., 2009a; Kesarcodi-Watson et al., 2009b; Romalde et al., 2014; Dubert et al., 2017). *V. neptunius* rapidly invades bivalve larvae tissues by entering them through the filtration feeding process. Its virulence is commonly associated with the production of thermolabile extracellular products with cytotoxic activity for fish and homeothermic animal cell lines (Dubert et al., 2016). Remarkably, vibriolysin-like protease VnpA and collagenase ColA were recently characterized as relevant virulence factors of *V. neptunius* since their production is required for full virulence in oyster larvae (*Ostrea edulis*) (Galvis et al., 2021).

It is well established that the production and utilization of siderophores is a key virulence factor for most vertebrate pathogenic bacteria (Kramer et al., 2020). However, although siderophore production would be a common feature of *Vibrio* pathogens affecting bivalves (Gómez-León et al., 2005; Mechri et al., 2017), its role in bacterial fitness and/or virulence is understudied in this type of bacterial pathogens. Our previous works demonstrated that *V. neptunius* produces a set of 9 amphibactin forms to overcome low-iron conditions. The gene cluster involved in its production and utilization was identified (genes *absABDEF* and *abtABCDE*) and the amphibactin outer membrane transporter gene *abtA* was characterized. It is noteworthy that *abtA* was regularly found in mollusc microbiota including some of the most devastating pathogens such as *V. coralliilyticus* and *V. tubiashii*. Interestingly, a *V. neptunius* Δ*absE* mutant (impaired to synthesize amphibactins) showed a weak but not null siderophore activity in cell free supernatants, which could imply the production of a second siderophore. Besides, the putative role of amphibactins in virulence for bivalves was not yet evaluated (Galvis et al., 2020).

In the present work the siderophore piscibactin was identified as the second siderophore produced by *V. neptunius*. Single and double *V. neptunius* mutants in biosynthetic genes of each siderophore system, piscibactin and amphibactin, were constructed and their role in virulence determined. In addition, the gene expression patterns of each siderophore were studied *in vitro* and *ex vivo*. The results showed that both siderophore systems, amphibactin and piscibactin, are expressed during infection, playing a key role in the virulence of *V. neptunius* for clams.

## Materials and Methods

### Bacterial Strains, Plasmids, and Media

The bacterial strains and plasmids used, as well as those derived from this study, are listed in **Table 1**. *V. neptunius* and *V. anguillarum* strains were grown at 25°C in Tryptic Soy Agar and Broth (Pronadisa, Madrid, Spain) supplemented with 1% NaCl (TSA-1 and TSB-1, respectively), as well as in M9 minimal medium supplemented with 0.2% Casamino Acids (Difco) (CM9) (Lemos et al., 1988). *Escherichia coli* strains were grown at 37°C in Luria-Bertani (LB) medium (Pronadisa) or LB supplemented with the appropriate antibiotics. Ampicillin sodium salt was used at 100 µg/mL, kanamycin 50 µg/mL and gentamycin 15 µg/mL (final concentrations).

**Table 1:** Strains and plasmids used in this study.

Strain or plasmid	Relevant characteristic(s)	Reference or source
*V. neptunius*		
PP-145.98	Wild type strain, isolated from *Ruditapes philippinarum* (larvae), Ap^r^	
FG109	PP-145.98 *absF* defective mutant, Ap^r^	This study
FG113	PP-145.98 *irp*2 defective mutant, Ap^r^	This study
FG115	PP-145.98 *absF* and *irp*2 defective mutant, Ap^r^	This study
*V. anguillarum*		
RV22	Wild-type serotype O2 strain isolated from diseased turbot (Spain)	(Lemos et al., 1988)
MB14	RV22 *vabF* defective mutant	(Balado et al., 2006)
MB67	RV22 *vabD* defective mutant	(Balado et al., 2018)
ML210	RV22 *vabD* and *frpA* defective mutant	submitted manuscript
*E. coli*		
DH5α	SupE4 Δ*lacU169* (Φ80 *lacZΔM15*) *hsd R17 recA1 endA1 gyrA96 thi-1 relA1*	Laboratory stock
S17-1-*λpir*	Tp^r^ Sm^r^ *recA, thi, pro, hsdR-M*+RP4: 2- Tc : Mu:Km Tn7 *λpir*	(Herrero et al., 1990)
Plasmids		
pWKS30	Low-copy-number cloning vector, Ap^r^	(Wang and Kushner, 1991)
pNidKan	Suicide vector derived from pCVD442, Km^r^	(Mouriño et al., 2004)
pHRP309	Low-copy lacZ reporter plasmid, mob Gm^r^	(Parales and Harwood, 1993)
pFG154	*proC* promoter (P*proC*) fused to promoterless *lacZ* gene in pHRP309, Gm^r^	This study
pFG156	*entD* promoter (P*entD*) fused to promoterless *lacZ* gene in pHRP309, Gm^r^	This study
pFG180	*abtA* promoter (P*abtA*) fused to promoterless *lacZ* gene in pHRP309, Gm^r^	This study
pFG166	*absE* promoter (P*absE*) fused to promoterless *lacZ* gene in pHRP309, Gm^r^	This study
pFG175	*abtA*(reverse) promoter (P*entD*-Reverse) fused to promoterless lacZ gene in pHRP309, Gm^r^	This study
pFG176	*araC1* promoter (P*araC1*) fused to promoterless *lacZ* gene in pHRP309, Gm^r^	This study
pFG188	*frpA* promoter (P*frpA*) fused to promoterless *lacZ* gene in pHRP309, Gm^r^	This study

### DNA Manipulations and Bioinformatics Tools

Total genomic DNA from *V. neptunius* PP-145.98 was purified with the InstaGene™ Matrix (BioRad, Hercules, California, CA, USA). PCR reactions were all carried out with Taq polymerase NZYTaq (Nzytech, Lisboa, Portugal) according to manufacturer protocol in a T-Gradient Thermal Cycler (Biometra, Göttingen, Germany). The extraction of DNA from agarose gels and purification of plasmid DNA were carried out using NucleoSpin Gel and a PCR clean-up kit (Macherey-Nagel, Düren, Germany) and a GeneJET Plasmid Miniprep Kit (Thermo-Fisher, Waltham, MA, USA).

The genome of *V. neptunius* PP-145.98 strain (accession number JAFHLB000000000) was screened, using antiSMASH 5.0 (Blin et al., 2019), for the presence of siderophore-related sequences. The NCBI services (http://ncbi.nlm.nih.gov) were used to consult the DNA and protein sequence databases with BLAST algorithm. Prediction of protein domains was carried out by using the Pfam protein families database (Finn et al., 2014). The sequences of housekeeping genes *ftsZ*, *gyrB*, *mreB*, *pyrH* y *recA* of bivalve pathogenic vibrios were downloaded from the NCBI database and used for multilocus sequence analysis (MLSA). Sequences of each gene were concatenated into a single 3880 bp sequence and aligned using the MUSCLE. The final phylogenetic tree was constructed based on concatenated sequences of the five housekeeping genes by maximum-likelihood (GTR+G model). Sequence alignments and phylogenetic tree were performed using MEGAX (v. 10.2.2) (Kumar et al., 2018).

### Construction of *absF* and *irp2* Mutants by Allelic Exchange

In-frame deletions of *absF* and *irp2* in *V. neptunius* PP-145.98 were constructed by using PCR amplification of two fragments of each gene and flanking regions that, when ligated together, would result in an in-frame (nonpolar) deletion. The oligonucleotides used to amplify the upstream and downstream ends of each gene are shown in **Table S1**. Once deleted alleles were constructed by sequential cloning of the PCR products into pWKS30 plasmid, they were liberated by digestion with *Not*I and *Apa*I and cloned into the suicide vector pCAR109 (Mouriño et al., 2004). The resulting plasmids (**Table 1**) were mated from *E. coli* S17-1-*λpir* into *V. neptunius* PP-145.98 and transconjugants with the plasmid integrated in the chromosome by homologous recombination, were selected on TSA-1 containing 50 mg/mL of kanamycin (resistance conferred by pNidKan) and 100 mg/mL of ampicillin (antibiotic to select *V. neptunius* PP-145.98). A second recombination event was obtained by selecting for sucrose (10%) resistance. This process led to the generation of the *V. neptunius* PP-145.98 single mutants Δ*absF* and Δ*irp2*, and the double mutant Δ*absF*Δ*irp2*, named FG109, FG113 and FG115, respectively.

### Growth Promotion and Siderophore Production Assays

Growth measurement of *V. neptunius* PP-145.98 strains was performed using 96-well microtiter plates. Each well contained 200 µL of CM9 medium (Lemos et al., 1988) supplemented with FeCl_3_ at 10 µM (PROBUS) or with the iron chelators ethylenediamine-di(o-hydroxyphenyl-acetic acid) (EDDA) at 5 µM or 2,2’–dipyridyl (dipyridyl) (Sigma) at 50 µM or 30 µM. Each well was inoculated with a 1:50 dilution of an overnight culture of the strain to be tested in TSB-1 at OD_600_ = 0.5. The plates were incubated at 25°C with shaking at 150 rpm. After 18 h of incubation, growth (OD_600_) was recorded in an iMACK Microplate reader (Bio-Rad). Bacterial cultures in CM9 with 30 µM 2,2’-dipyridyl and an OD_600_ ≈0.6 (after 6 h of incubation) were used to measure siderophore production using the chrome azurol-S (CAS) liquid assay (Schwyn and Neilands, 1987). Equal volumes of each cell free supernatant and CAS reagent were mixed and absorbance at 630 nm (A_630_) was measured in a UV-VIS spectrophotometer (Hitachi) after 15 min of incubation at room temperature.

### Cross-Feeding Assays

The biological activities of the supernatants produced by the parental and mutant strains were determined by cross-feeding experiments. We tested the ability of culture supernatants from *V. neptunius* PP-145.98 mutants defective in piscibactin and amphibactin synthesis to cross-feed different indicator strains defective in the synthesis and/or transport of piscibactin.

To test whether *V. neptunius* wild type or derivative mutants produce piscibactin, a cross-feeding assay was conducted using two *V. anguillarum* mutants derived from RV22 strain that lack siderophore synthesis: RV22*ΔvabD*, a single mutant (strain MB67) that does not produce siderophores and that it is able to use piscibactin as iron source since it carries the piscibactin outer membrane transporter FrpA; and RV22*ΔvabDΔfrpA*, a double mutant that does not use piscibactin since it has inactivated the piscibactin transporter FrpA. Indicator strains were inoculated into CM9 plates as follows: 0.5 mL of an overnight culture in TSB-1 at an OD_600_ = 0.5 were mixed with 5 mL of CM9 medium containing 0.5% agarose and 2,2’-dipyridyl 100 µM, a concentration close to the Minimal Inhibitory Concentration (MIC) and at which growth halos can be easily visualized (Balado et al., 2006). The *V. neptunius* strains to be tested for piscibactin production were cultured in TSA-1 plates and the cells were harvested with a sterile loop and placed on the surface of the plates inoculated with the *V. anguillarum* strains. A piscibactin producer *V. anguillarum* strain (RV22 *ΔvabF*) was used as control. The presence of growth halos of the *V. anguillarum* indicator strains around cells of *V. neptunius* after overnight incubation at 25°C was indicative of piscibactin production.

### RNA Purification and RT-PCR

The organization of amphibactin genes into operon(s) was tested by reverse transcription PCR. *V. neptunius* PP-145.98 was grown until exponential phase (ca. OD_600_ = 1) in 10 mL CM9 medium containing 2.5 µM EDDA. Cells were pelleted by centrifugation at 10,000 × g for 2 min and total RNA was isolated with Trizol (Invitrogen) following the manufacturer’s recommendations. RT-PCR was performed with 1 µg RNA pre-treated with RQ1 RNase-Free DNase (Promega) by using the M-MLV reverse transcriptase (Invitrogen). A primer located at the 3’-end of *absA* gene was used to obtain a cDNA that was used as template for subsequent PCR reactions targeted in *abtE* (PCR2) and in the region between *abtC* and *abtD* (PCR1), *absE* and *absF* (PCR3) and between *abtA* and absB (PCR4) (**Table S1**). A negative control reaction was performed with total RNA treated with DNase without M-MLV reverse transcriptase to confirm the lack of genomic DNA contamination in each reaction mixture. The PCR positive control reaction was done using 100 ng of genomic DNA as template.

### *lacZ* Transcriptional Fusions and β-Galactosidase Assays

The DNA fragments corresponding to the amphibactin and piscibactin promoter regions of *V. neptunius* PP-145.98 were amplified by PCR. The PCR products spanned from the first nucleotides of the coding sequence to ca. 700-900 bp upstream sequence. These putative promoter regions were fused to a promoterless *lacZ* gene in the low-copy-number reporter plasmid pHRP309 (Parales and Harwood, 1993). Transcriptional fusions of genes *abtC* and *abtA* for amphibactin, and *araC1* and *frpA* for piscibactin were constructed. The resulting transcriptional fusion constructs, *abtC*::*lacZ* (pFG156), *abtA*::*lacZ* (pFG180), *arac1*::*lacZ* (pFG176) and *frpA*::*lacZ* (pFG188), were mobilized from *E. coli* S17-1-λpir into *V. neptunius* PP-145.98 by conjugation. *V. neptunius* PP-145.98 derivatives carrying the transcriptional fusions were grown in CM9 at 25°C under conditions of iron availability (CM9 with 10 µM Fe_2_(SO_4_)_3_) and iron deficiency (CM9 with 50 µM 2,2’-dipyridyl). For the *ex vivo* assay *V. neptunius* PP-145.98 derivatives carrying the promoter-*lacZ* fusions were grown at 25°C in the hemolymph of commercial mussels (*Mitylus galloprovincialis*) under normal or excess iron conditions. Hemolymph was collected from approximately 30 mussels by puncture of the adductor anterior muscle, then centrifuged at 4,000 rpm for 10 min and filtered through a nitrocellulose membrane with a pore size of 0.25 µm. The β-galactosidase (LacZ) activity in CM9 cultures and hemolymph were measured by the method of Miller (Miller, 1992). Results showed are means of three independent experiments, each one measured in triplicate. Statistical significance of differences was determined using *t*-test. *P*-values were considered significant when *P* was <0.05.

### Virulence Assays

Experimental infections in healthy clams (*Ruditapes philippinarum*) were performed with the wild-type and mutants of *V. neptunius* PP-145.98. For acclimatization the clams (mean size of 10 mm) were kept for 2 days in a seawater tank at 20°C with continuous aeration. The wild-type strain and the mutants Δ*absF*, Δ*irp2* and Δ*absF*Δ*irp2* of *V. neptunius* PP-145.98 were cultured in CM9 medium with 2,2’-dipyridyl 20 µM for 12 h at 25°C to achieve an OD_600_ = 0.6. The infection was carried out by bath as follows: groups of 50 clams were introduced in 50 mL of seawater containing the bacterial strain to be tested at a final concentration of 10^6^ cfu/mL, and incubated for 24 h. The clams were then washed twice with abundant seawater and placed in containers with 200 mL of seawater with aeration. The assay was performed by duplicate and a group of clams under the same conditions but without bacterial inoculation was used as negative control. Pathogenicity was evaluated after 96 h and was expressed as a percentage of survival. Mortalities were recorded daily, and the statistical significance of differences in percentage of survival for the different *V. neptunius* strains was determined using the Kaplan–Meier method with the Mantel–Cox log-rank test using SPSS (version 20; IBM SPSS Inc., Chicago, IL, USA). *P*-values were considered significant when *P* was <0.05, <0.01 and <0.001.

Virulence assays on fish were performed using turbot (*Scophthalmus maximus*) fingerlings weighting 5 g on average. The fish were divided into groups of 10 animals. All groups, one per strain tested and a control group, were maintained in 50 L seawater tanks at 18°C with continuous aeration and water recirculation. The wild-type strain and mutants were grown in CM9 medium for 12 h at 25°C to reach an OD_600_ = 0.6. The inoculum used was a 10-fold dilution of this suspension in saline solution (0.85% NaCl in distilled H_2_O). Fish were inoculated intraperitoneally (ip) with 100 µL of bacterial suspensions. A control group was inoculated with 100 µL of saline solution. Mortalities were recorded daily for 15 days after injection. All animal experimentation protocols used in this study were reviewed and approved by the Animal Ethics Committee of the University of Santiago de Compostela (protocol id. 15004/14/003).

### Piscibactin Detection by Mass Spectrometry

The presence of piscibactin was studied following the SPE-HLB/HPLC-MS methodology previously described (Souto et al., 2012; Balado et al., 2018). Briefly, *V. neptunius* Δ*absF* was grown in CM9 medium supplemented with 30 µM 2,2’–dipyridyl at 25°C under continuous shaking (150 rpm) for 24 h. Once achieved an OD_600_ = 1, the bacterial culture was centrifuged at 10,000 × *g* for 10 min (Beckman J-21 High Speed Centrifuge) and filtrated through a 0.45-μm pore size membrane. 800 mL of the resultant cell-free supernatant were treated with 17 mg of FeCl_3_, incubated at 4°C for 12 h, and concentrated under reduced pressure to 300 mL. Half of this volume was fractionated using an Oasis^®^ hydrophilic lipophilic balance (HLB) cartridge (Waters) (35 cm^3^, 6 g), previously conditionated with 60 mL of acetonitrile (ACN, solvent B) and deionized water (H_2_O, solvent A), in three batches of 75 mL. Each batch was fractionated with 30 mL of the following solvent mixtures: 1:0, 7:3, 1:1, 3:7, and 0:1 of A:B (v/v) obtaining the fractions VNΔ*absF*H1-5 respectively. VNΔ*absF*H3 was analysed by HPLC/HRMS in a HPLC Acela (Thermo) coupled to a PDA and HRMS (LQT-Orbitrap Discovery) detector in full positive ion using the Atlantis dC18 (100 x 4.6 mm, 5 µm) column (Waters) and the following method (solvent A: H_2_O, solvent B: ACN): 35 min from 10% to 100% of B, 5 min isocratic at 100% of B and 10 min from 100 to 10% of B at 1 mL min^-1^. The results showed the presence of ferri-piscibactin in the chromatographic peak with a R_t_=10.69 min displaying the [M+H]^+^ adduct at *m/z* 507.0032 (calcd. for C_19_H_21_N_3_O_4_S_3_Fe^+^ *m/z* 507.0038) and the absorbance maxima at 227, 256, 307, and 388 nm in its UV spectrum.

## Results

### *V. neptunius* Genome Harbors a Version of the High Pathogenicity Island (irp-*HPI*) Encoding Piscibactin Biosynthesis and Transport

**Figure 1:**
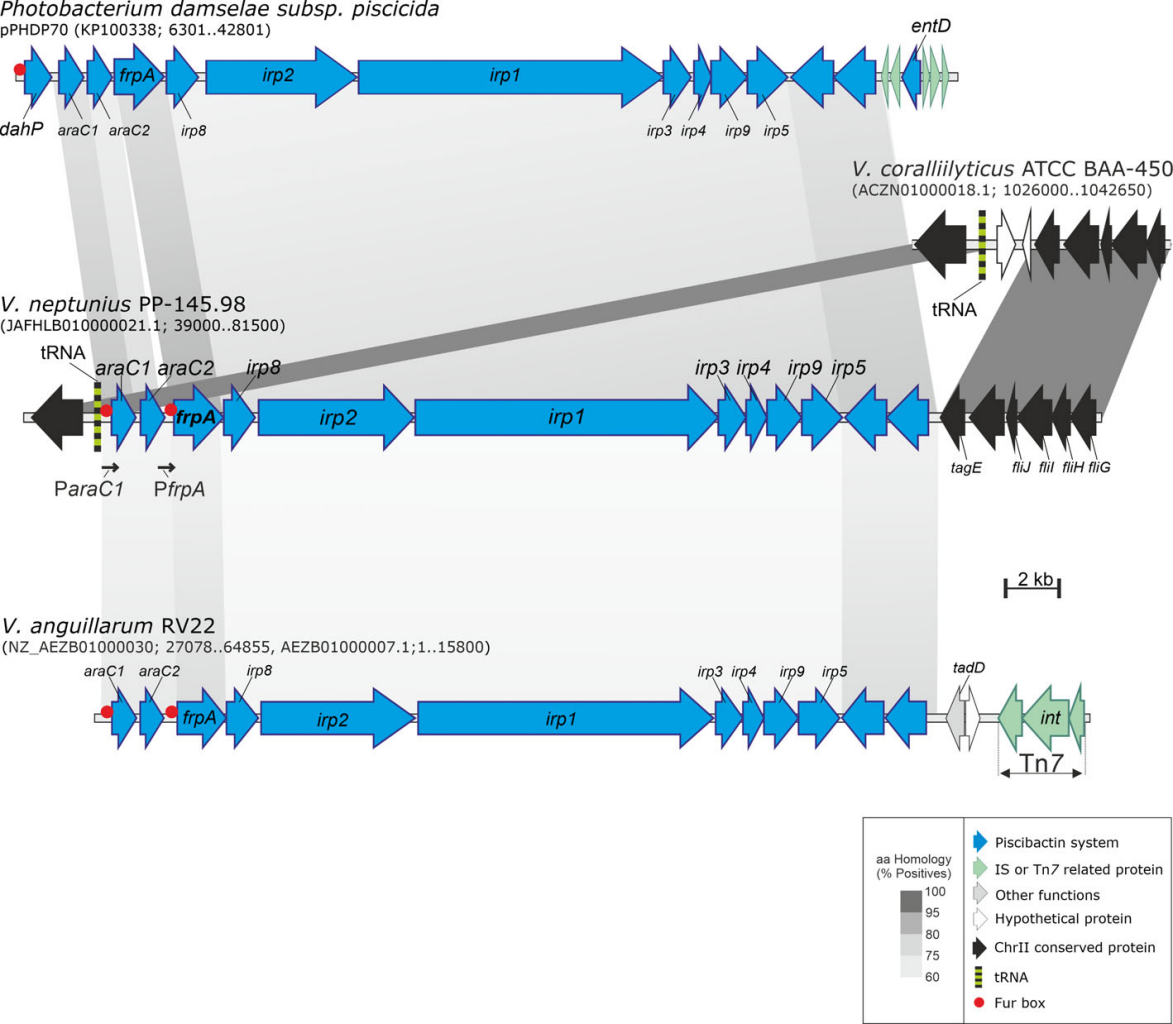
Genetic map of the gene cluster encoding the piscibactin system in *V. neptunius.* The piscibactin genes, which are part of *V. angullarum* RV22 chromosome II and the plasmid pPHDP70 of *P. damselae* subsp. *piscicida* are included for comparative purposes. Grey blocks indicate percentages of similarity in the proteins sequence.

*In silico* analysis of *V. neptunius* PP-145.98 genome sequence (NZ_JAFHLB010000000) led to the identification of a gene cluster with high homology to *irp* genes previously identified in *P. damselae* subsp. *piscicida* and several Vibrios. These genes are harbored in the High Pathogenicity Island named *irp*-HPI and confer the ability to produce and use the siderophore piscibactin (Osorio et al., 2015) (**Figure 1**). The genomic island *irp*-HPI of *V. neptunius* (*irp*-HPI_Vnep_) is a DNA region of approximately 33 kb that is located in the chromosome II between a tRNA gene and the flagellum operon (**Figure 1**). *irp*-HPI_Vnep_ shares identical structure and genetic organization with the piscibactin gene cluster present in *P. damselae* subsp. *piscicida* (KP100338) and *V. anguillarum* RV22 (AEZB01000000) (Souto et al., 2012; Osorio et al., 2015; Balado et al., 2018). In addition, the *irp*-HPI_Vnep_ predicted products showed an amino acid similarity between 71 and 82% with the *irp* cluster ortholog from *P. damselae* subsp. *piscicida*, and between 54% and 65% with those from *V. anguillarum* RV22 (**Figure 1** and **Table S2**). Although *V. neptunius irp*-HPI shows higher similarity to *P. damselae* subsp. *piscicida* genomic island than to *V. anguillarum*, *V. neptunius* and *V. anguillarum irp*-HPI genomic islands share a series of characteristics. Among them, they do not include a *dahP* and probable Fur box motifs are found upstream of *araC1* and *frpA* (**Figure 1**). To ascertain whether the *irp*-HPI_Vnep_ genes are expressed, a series of retro-transcriptase nested PCR reactions were performed. A PCR targeted to *araC1* gene showed the existence of an mRNA covering from *araC1* to *irp5* (data not shown) indicating that the gene cluster is transcribed in a polycistronic mRNA. The same result was found for the piscibactin gene cluster of *P. damselae* subsp*. piscicida* and *V. anguillarum* (Balado et al., 2018).

**Figure 2:**
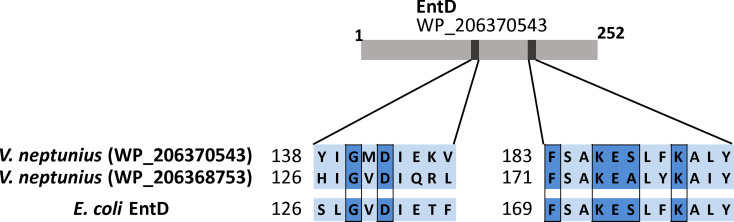
Highly conserved residues of the type-II PPTases shared between the two *V. neptunius* predicted proteins and the EntD of *E. coli*.

The non-ribosomal peptide synthetases (NRPSs) require a phosphopantetheinyl transferase (PPTase) to be active (Orikasa et al., 2006) but *irp*-HPI genomic islands do not include this function (Osorio et al., 2015; Balado et al., 2018). *In silico* search showed the existence of up to two probable PPTases (loci WP_206370543.1 and WP_206368753.1), whose predicted products are homologous to several groups of PPTases containing all conserved residues required to be functional (**Figure 2**) (Lambalot et al., 1996; Liu et al., 2005).

### Inactivation of *irp*-HPI Genes Reduces the Growth Ability of *V. neptunius* Under Iron Restriction

**Figure 3:**
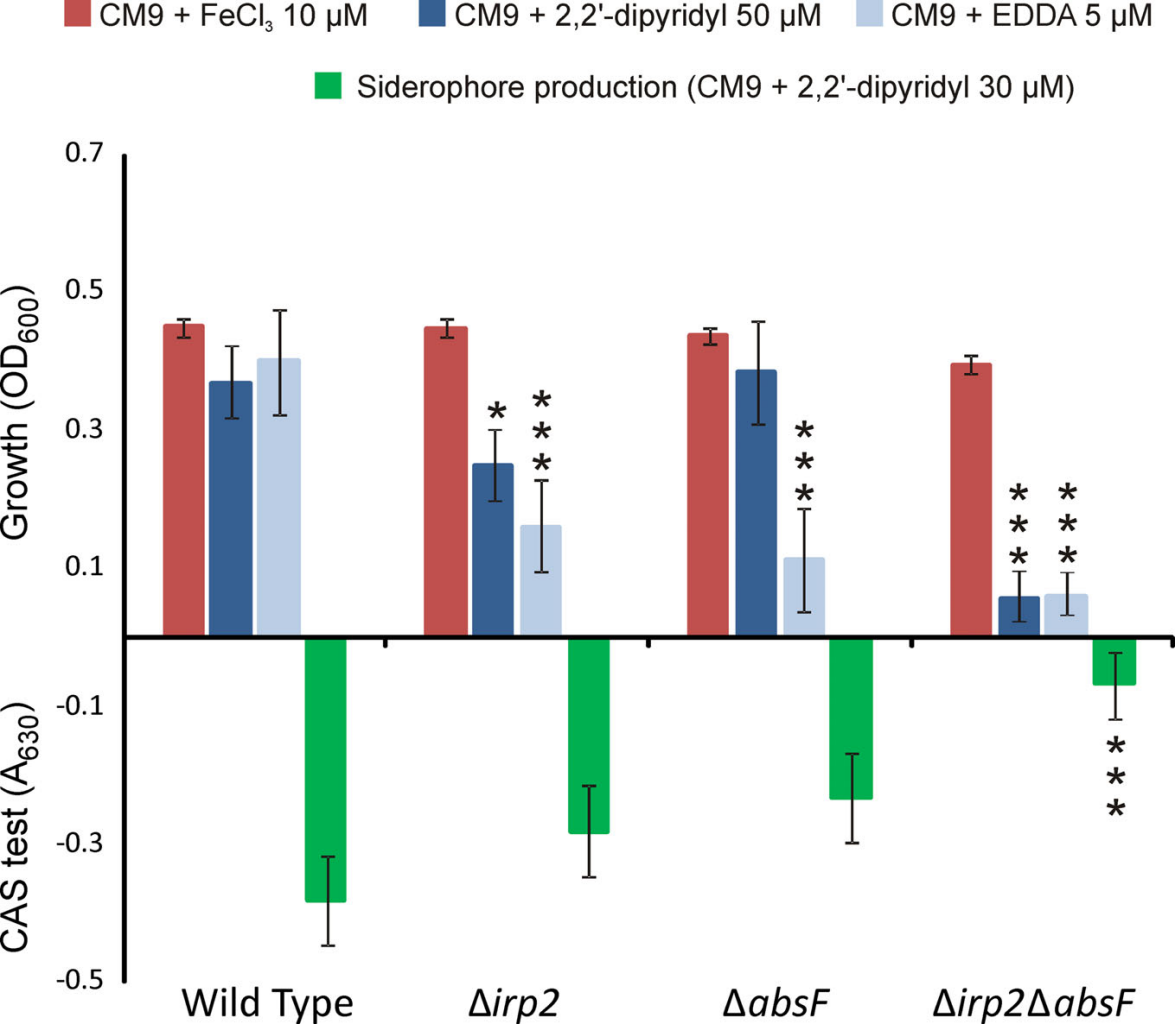
Growth levels under low and high-iron availability and siderophore production (CAS assay) of *V. neptunius* PP-145.98 wild type and its derivative mutants Δ*absF*, Δ*irp2* and Δ*irp2-*Δ*absF*. Asterisks denote statistically significant differences with the wild type strain (*P < 0.05 and ***P < 0.001).

To test whether *irp*-HPI genes mediate siderophore synthesis in *V. neptunius* PP-145.98, single and double mutants in *irp2* and *absF* genes were constructed. *irp2* and *absF* encode NRPSs required for piscibactin and amphibactin synthesis, respectively (Souto et al., 2012; Galvis et al., 2020). The growth capacity and siderophore production of each mutant were evaluated under different iron availability conditions and their phenotypes were compared to the parental strain (**Figure 3**). *V. neptunius* wild type strain and all derivative mutants showed indistinguishable growth ability under iron excess (CM9 plus 10 μM ferric chloride). By contrast, under iron-restricted conditions some differences were observed. Iron-restricted conditions were achieved by adding to the minimal medium CM9 the strong iron chelating agent EDDA or the weaker chelator dipyridyl (McMillan et al., 2010). The *V. neptunius Δirp2ΔabsF* double mutant showed a MIC of EDDA of 5 µM while the MIC of dipyridyl was 50 µM. By contrast, the growth ability of the wild type strain under weak or strong iron-restriction (CM9 plus dipyridyl 50 μM or EDDA 5 μM, respectively) was almost the same as under iron-excess. Interestingly, some differences were observed between growth of *ΔabsF* and *Δirp2* single mutants under iron-restriction. Addition of the weak iron chelator dipyridyl at 50 μM significantly reduced the growth of the *Δirp2* mutant, but it did not affect the growth of the *ΔabsF* mutant. Under strong iron-restricted conditions both single mutants showed reduced growth, ca. 60% in the Δ*irp2* mutant and ca. 70% in the *ΔabsF* mutant. Finally, siderophore activity present in cell free supernatants, measured by the CAS assay, after growth of each *V. neptunius* strain in non-restrictive conditions (CM9 plus dipyridyl at 30 μM) showed that siderophore production was almost abolished in the Δ*irp2*Δ*absF* double mutant. By contrast, although single inactivation of *Δirp2* or *ΔabsF* showed lower siderophore content in supernantants compared to wild type, the differences were not statistically significant (**Figure 3**).

**Figure 4:**
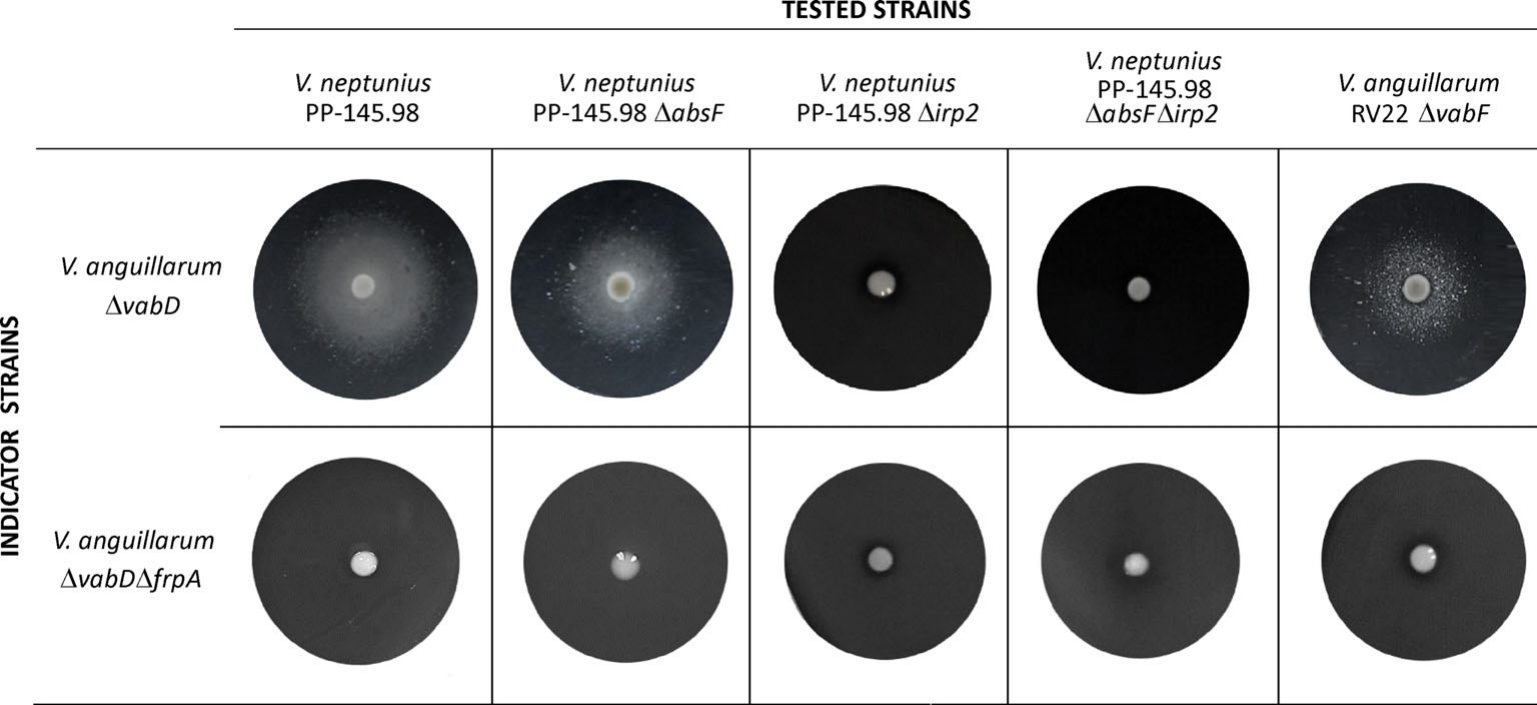
Cross feeding assay to test the production of piscibactin by *V. neptunius 145.98* wild type strain and its derivative Δ*absF* and Δ*irp2* single mutants and Δ*irp2*Δ*absF* double mutant. Indicator strains were inoculated within the CM9 plates containing 100 µM of 2,2’-dipyridyl. Tested strains were placed as a loop full of bacterial biomass on the agar surface. The presence of a growth halo shows that the indicator strains can use the siderophores produced by the tested strains to overcome the iron limitation.

To test whether *V. neptunius* wild type and mutant strains produced piscibactin, a series of cross-feeding assays were performed. A *V. anguillarum* FrpA^+^ (Δ*vabD*) strain that has a functional piscibactin transporter FrpA, (can use piscibactin as iron source) and a FrpA^–^ (*ΔvabDΔfrpA* double mutant) that cannot use piscibactin since it lacks FrpA, were used as indicator strains. Both indicator strains do not produce any siderophore due to the inactivation of the PPTase gene *vabD* (Balado et al., 2018). The results showed that the *V. neptunius* wild-type strain and the *ΔabsF* single mutant promoted the growth of *V. anguillarum* FrpA^+^, but they did not cross-feed the FrpA^–^ indicator strain. Congruently, neither *V. neptunius Δirp2* nor *Δirp2ΔabsF* mutants induced the growth of FrpA^+^ (**Figure 4**).

**Figure 5:**
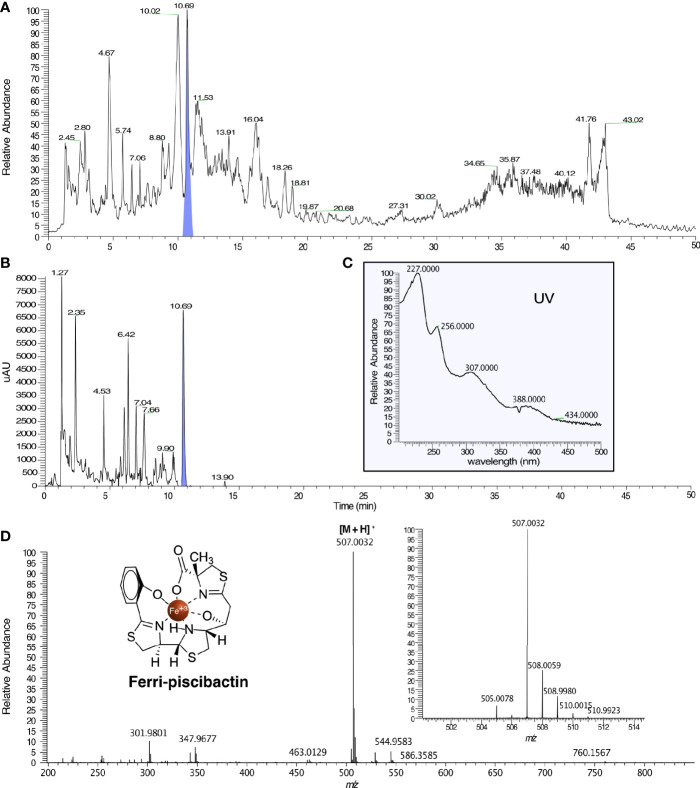
HPLC/HRMS analysis for the detection of ferri-piscibactin in the fraction VNΔabsFH3, from the *V. neptunius* Δ*absF* mutant, eluted with 1:1 H_2_O:ACN from a HLB cartridge. **(A)** Total Ion Current (TIC) chromatogram of VNΔabsFH3 in which ferri-piscibactin was detected at R_t_ = 10.69 min, highlighted in blue. **(B)** HPLC-DAD chromatogram of VNΔabsFH3 in which ferri-piscibactin was detected at R_t_ = 10.69 min, highlighted in blue. **(C)** UV spectrum of the peak at R_t_ = 10.69 min, where ferri-piscibactin was detected, showing the absorption maxima at 207, 256, 307 and 388 nm. **(D)** (+)-HRESIMS of the peak at R_t_ 10.69 min, where ferri-piscibactin was identified, displaying: *m/z* 507.0032 (calcd. for C_19_H_21_N_3_O_4_S_3_Fe^+^, *m/z* 507.0038), expanded region of MS in the range *m/z* 501-514, showing the presence of the characteristic Fe isotopic distribution, and structure of ferri-piscibactin.

To prove that *V. neptunius* 145.98 synthesizes piscibactin in addition to amphibactin, cell free supernatants from *ΔabsF* single mutant were studied following the SPE-HLB/HPLC-HRMS methodology previously described by our research group (Souto et al., 2012). Cell-free supernatant was treated with FeCl_3_, to obtain the stable ferri-siderophores, and fractionated using an HLB cartridge. HPLC/HRMS analysis of the CAS-positive fraction VNΔ*absF*H3, eluted with 1:1 of ACN:H_2_O, confirmed the presence of ferri-piscibactin in the chromatographic peak at R_t_=10.69 min (**Figures 5A, B**) by comparison of its MS and UV spectra with the previously described data (Souto et al., 2012). Specifically, the mass spectrum (MS) of this peak showed the [M + H]^+^ adduct at *m/z* 507.0032 (calcd. for C_19_H_21_N_3_O_4_S_3_Fe^+^, *m/z* 507.0038) along with the isotopic distribution of Fe (Mr = 54, 56, 57, 58; ratio 0.6:9.2:0.2:0.02) (**Figure 5D**); while the UV spectrum displayed the absorbance maxima at 227, 256, 307, and 388 nm (**Figure 5C**).

All these results demonstrate that each one of the *V. neptunius* single mutants (*Δirp2* or *ΔabsF*) produce one unique siderophore when cultivated under iron-restricted conditions. Thus, while *V. neptunius Δirp2* mutant produces only amphibactin and *ΔabsF* produces only piscibactin, the *Δirp2ΔabsF* double mutant does not produce any of these siderophores.

### Piscibactin and Amphibactin Significantly Contribute to *V. neptunius* Virulence for Clams

**Figure 6:**
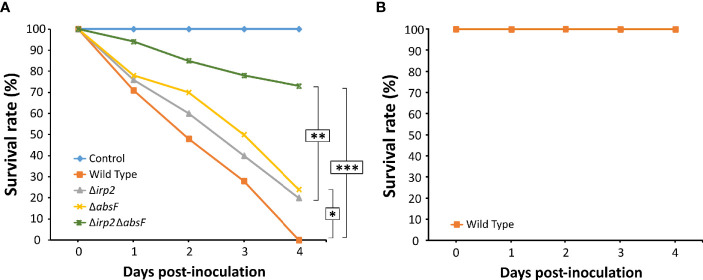
Survival curves after infection challenge in clams (*Ruditapes philippinarum*) **(A)** and turbot **(B)** with *V. neptunius* wild-type strain and its derivatives Δ*absF*, Δ*irp2* and Δ*irp2*Δ*absF* mutants. Asterisks denote statistically significant differences between strains (*P < 0.05; **P < 0.01 and ***P < 0.001).

To study the role of piscibactin and/or amphibactin production in virulence of the bivalve mollusc pathogen *V. neptunius*, experimental infection challenges were performed. For this purpose, groups of 50 clams larvae were inoculated with the *V. neptunius* wild type or with one of the mutants. A 100% mortality was obtained in the group inoculated with *V. neptunius* wild-type strain 4 days after the challenge (**Figure 6A**) and no mortality was observed in the non-challenged control group. Interestingly, mortality registered after inoculation with *ΔabsF* or *Δirp2* single mutants decreased up to 75-80% (**Figure 6A**), which represents statistically significant differences with respect to the group inoculated with the wild type strain. Most notably, mortality was strongly reduced in the clams group inoculated with the *ΔabsFΔirp2* double mutant, where almost 80% of larvae survived to the challenge (**Figure 6A**). To test the possible virulence of *V. neptunius* wild type for fish, an experimental infection was done also in fish, using turbot fingerlings. In this case, there was not registered any mortality (**Figure 6B**), which showed that *V. neptunius* PP-145.98 wild type strain is pathogenic only for bivalve molluscs.

### Amphibactin Gene Cluster Transcriptional Organization

**Figure 7:**
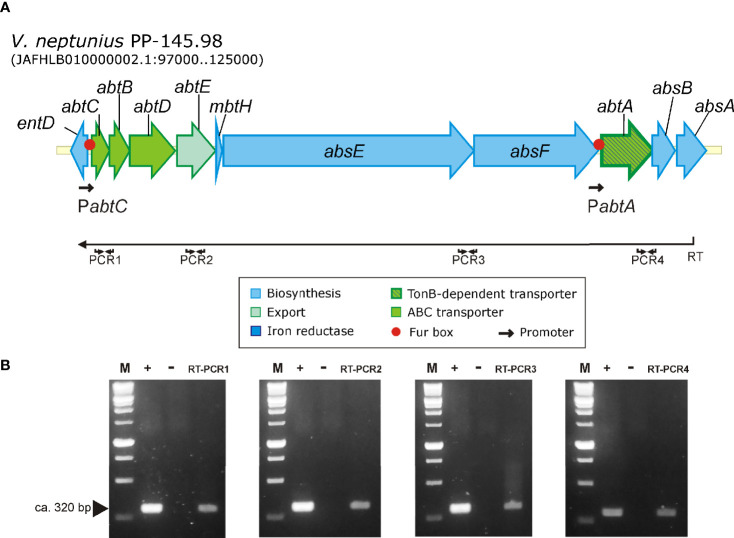
Results of RT-PCR reactions designed to analyze the transcription of the amphibactin gene cluster in *V. neptunius* PP-145.98. **(A)** Genetic map of the gene cluster encoding the amphibactin in *V. neptunius* PP-145.98. **(B)** An appropriate primer (Primer RT, **Table S1**), located at the 3’-end of *absA* gene, was used to obtain a cDNA (denoted as RT in the figure) that was then used as template for 4 PCR reactions targeted between *abtC* 3’ -end and 5’ -end *abtD* (PCR1), *abtE* (PCR2), between *absE* 3’ -end and *absF* 5’ -end (PCR3), and between *abtA* 3’ -end and *absB* 5’ -end (PCR4). M, size marker from 100 to 1,000 bp; +, positive control PCR using genomic DNA as template; −, negative control PCR using total RNA without reverse transcriptase. RT-PCR1-4 shows the amplification result of the 4 different RT-PCRs.

Although amphibactin is one of the most abundant siderophores in the seawater (Boiteau et al., 2016; Gauglitz et al., 2021), transcriptional organization of amphibactin genes was not studied to date. To define the transcriptional organization of amphibactin genes, a series of reverse transcriptase PCR reactions (RT-PCRs) were performed. Results are shown in **Figure 7**. Since all amphibactin genes are encoded in the same DNA strand (**Figure 7A**), a reverse transcriptase reaction was done by using a primer targeted on *absA*, the last gene of the cluster (**Figure 7A**). Positive amplification was found in a PCR targeted into the *abtC* gene using previously obtained cDNA as template. This result clearly shows that amphibactin genes are transcribed into a polycistronic mRNA spanning from *abtC* to *absA* genes. Consequently, they must be transcribed from a divergent promoter located in the *entD*-*abtC* intergenic region (**Figure 7B**). Nevertheless, this result does not rule out the possibility that additional promoters exist within the gene cluster driving independent transcription of some genes. *In silico* analysis of the intergenic sequences of the amphibactin cluster suggests the existence of two putative FUR box motifs: one located 113 bp upstream of *abtC* start codon (GCAAACCATTTTCATTTGC) and another one located 83 bp upstream of *abtA* (*absF*-*abtA* intergenic region) (GATAACCATTATTATCATTAGC) (**Figure 7A**). AbtA was previously characterized as the TonB-dependent outer membrane transporter required for ferri-amphibactin internalization (Galvis et al., 2020). These Fur box motifs showed an identity of 58 and 79%, respectively, to the FUR box motif consensus sequence of *E. coli* (GATAATGATAATCATTATCATTATC) (Ochsner and Vasil, 1996; Payne et al., 2016). Thus, the existence of a promoter upstream of *abtA* controlling the expression of amphibactin transporter will be further studied.

### Both Siderophore Systems, Amphibactin and Piscibactin, Are Significantly Expressed When *V. neptunius* Is Cultivated Under Iron Deficiency

**Figure 8:**
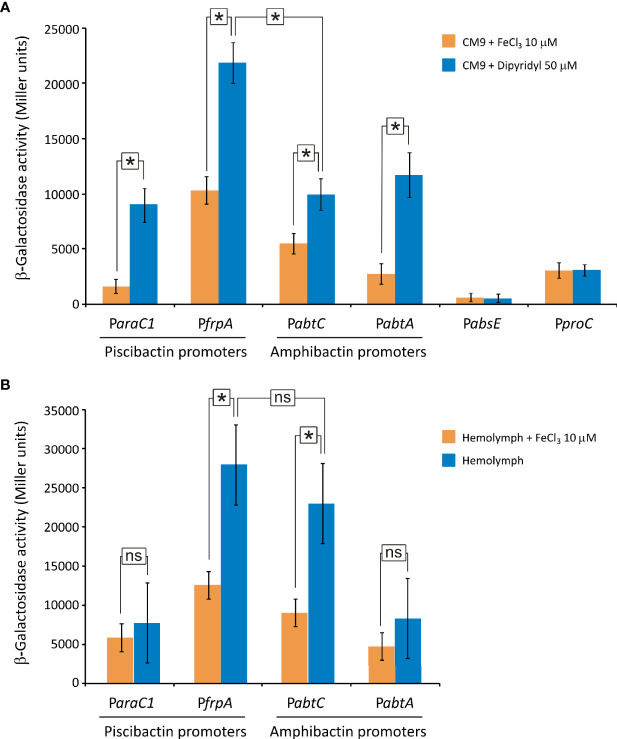
Transcriptional activity (β-galactosidase units) of piscibactin (P*araC1* and P*frpA*) and amphibactin (P*abtC* and P*abtA*) promoters carried out using *lacZ* fusions. Expression assay was performed *in vitro* by cultivating *V. neptunius* PP-145.98 in CM9 minimal medium supplemented with FeCl_3_ 10 µM (iron excess) or 2,2’-dipyridyl 50 µM (iron deficiency) **(A)** and *ex vivo* by cultivating bacteria in mussel hemolymph **(B)**. P*abtA* promoter cloned in reverse orientation upstream of the *lacZ* gene was used as a negative control. A *lacZ* fusion with the constitutive promoter *proC* (P*proC*) was used as control. Asterisks indicate statistically significant differences between bars (*P* < 0.05); ns, no statistically significant differences. Error bars denote standard deviations.

To evaluate the transcription levels of both amphibactin and piscibactin siderophore systems, the promoter regions of each siderophore gene cluster were cloned into the promoterless plasmid pHRP309 upstream of the *lacZ* gene. Resulting plasmids were mobilized into *V. neptunius* PP-145.98 wild type strain and the transcription levels of each promoter were evaluated *in vitro* and *ex vivo* (**Figure 8A**). To identify amphibactin promoters, fusions of the putative promoters *abtA*, *abtC*, and the sequence upstream of *absE* were evaluated by measuring β-galactosidase activity. These regions were named P*abtA*, P*abtC and* P*absE*, respectively (**Figure 8A**). On the other hand, since P*araC1* and P*frpA* were previously characterized as the promoters that control piscibactin expression in *V. anguillarum* (Balado et al., 2018), *lacZ* fusions of the *V. neptunius* sequences upstream of *frpA* and *araC1* (P*frpA* and P*araC1*) were also obtained. The P*abtA* promoter cloned in reverse orientation was used as negative control and its transcriptional levels were almost undetectable under all the growth conditions tested (data not shown).

Piscibactin promoters (P*araC1* and P*frpA*) as well as amphibactin promoters P*abtC* and P*abtA* were assayed under low-iron availability *in vitro* (CM9 with 50 μM dipyridyl). The results show that all these promoters displayed high *β*-galactosidase activities (**Figure 8A**). However, the sequence upstream of *absE* showed an almost undetectable *β*-galactosidase activity under all the conditions tested **(** **Figure 8A**). Thus, the presence of an independent promoter upstream of the biosynthetic gene *absE* was discarded. It is noteworthy that while piscibactin promoter P*frpA* achieved an activity of ca. 22,000 U under low iron availability, the activity of P*abtC*, P*abtA* and P*araC1* were 50% lower (ca. 10,000 U). These results would suggest that, under iron deficiency, the expression of piscibactin genes would be predominant. As expected, the addition of 10 μM FeCl_3_ to the culture medium greatly reduced (>40%) the transcriptional activity of amphibactin and piscibactin promoters, indicating that the two siderophore systems are significantly less expressed when iron availability increase (**Figure 8A**). The transcriptional activity of the constitutive promoter *proC* was independent of iron concentration.

To assess the expression of the siderophore systems during bacteria-bivalve interaction, an *ex vivo* assay was performed by the incubation of *V. neptunius lacZ* fusion carrier strains in mussel hemolymph (*Mytilus galloprovincialis*). Interestingly, the expression pattern registered *ex vivo* (**Figure 8B**) showed some differences with that observed *in vitro* (**Figure 8A**). Specifically, the transcriptional activity of the amphibactin promoter P*abtC* was 2-fold higher when incubated in hemolymph, achieving ca. 25,000 U. Thus, while the piscibactin promoter P*frpA* showed *in vivo* a notable higher expression than amphibactin promoter P*abtC*, there were no statistically significant differences between them *ex vivo*. Addition of 10 μM FeCl_3_ to the hemolymph also significantly reduced transcriptional activity of both siderophore systems, which suggests that iron limitation must be a signal that bacteria detect when entering the host (**Figure 8B**).

### Piscibactin and Amphibactin Are Present in Most Pathogenic *Vibrio* Species of the Corallilyticus Clade

**Figure 9:**
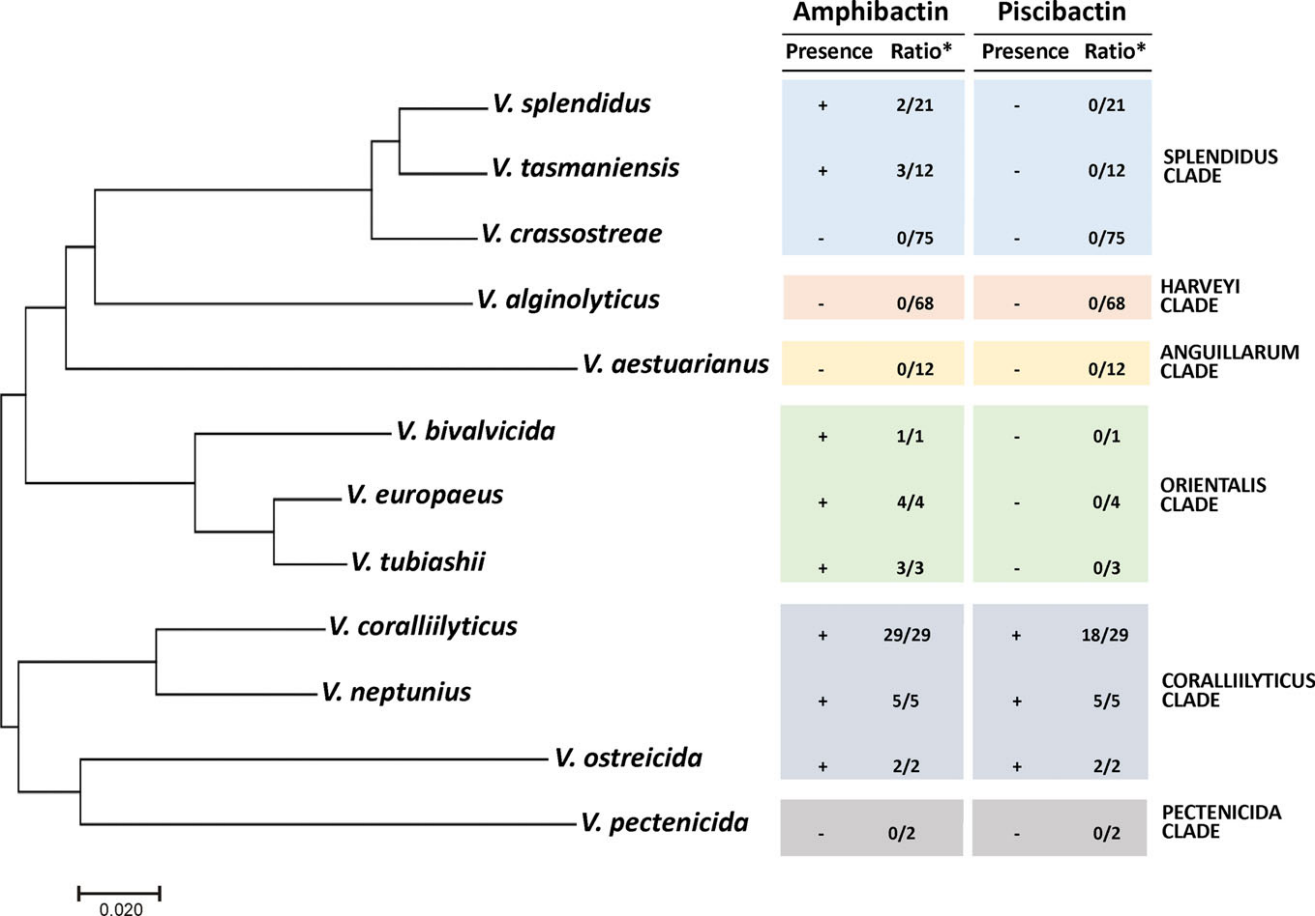
Distribution of amphibactin and piscibactin siderophore systems in *Vibrio* species responsible for outbreaks in bivalves hatcheries. Presence (+) or absence (-) of complete piscibactin/amphibactin gene cluster determined by Blast search in WGS database indexed in the NCBI database using the nucleotide sequences of *V. neptunius* PP-145.98 as query. The ratio (*) indicates the number of genomes of a *Vibrio* sp. harboring close homologues with respect to the total number of genomes analyzed of that species. Phylogenetic tree was constructed by maximum-likelihood (GTR+G model) with the concatenated sequences of the five housekeeping genes *ftsZ*, *gyrB*, *mreB*, *pyrH* and *recA*.

To evaluate the distribution of amphibactin and piscibactin siderophore systems in species of *Vibrio* with importance in bivalve aquaculture, a total of 234 genomes of *Vibrio* species belonging to the Coralliilyticus, Anguillarum, Harveyi, Orientalis, Pectenecida and Splendidus clades were tested *in silico* for the presence of complete amphibactin and piscibactin gene clusters. These species were selected since they are commonly reported as bacteria associated with mortality events in larvae and spats of bivalves hatcheries and their virulence for bivalves is well-established (Beaz-Hidalgo et al., 2010; Dubert et al., 2017). The results (**Figure 9** and **Table S3**) showed that most *Vibrio* genomes belonging to the Coralliilyticus clade harbor complete gene clusters encoding amphibactin and piscibactin siderophore systems. All *V. neptunius*, *V. ostreicida* and *V. coralliilyticus* genomes available in GenBank harbor close homologues of amphibactin genes, sharing identical structure and an average identity of 99%, 67% and 84%, respectively, with those found in *V. neptunius* PP-145.98 strain. The genomic island *irp*-HPI is present in all *V. neptunius* and *V. ostreicida* genomes sequenced and also in most *V. coralliilyticus* (18 of the 29). These results altogether suggest that most *Vibrio* species that belong to the Coralliilyticus clade harbor close homologues of both amphibactin and piscibactin siderophore systems. In addition, the amphibactin system is also widespread among vibrios of the Orientalis clade (*V. bivalvicida*, *V. europeus* and *V. tubiashii*). Finally, among vibrios of the Splendidus clade, 9.5% of *V. splendidus* and 25% of *V. tasmaniensis* genomes also harbor amphibactin genes.

## Discussion

Bacterial virulence is context-dependent and mainly depends on the interaction among diverse virulence factors functioning at the same time or sequentially when bacteria enter the host (Won and Park, 2008). The main virulence factors characterized to date in bacteria pathogenic for marine bivalve molluscs are those related to motility, chemotaxis and, in a major extent, to extracellular products (de O Santos et al., 2011; Kimes et al., 2012; Ushijima et al., 2014; Ushijima et al., 2018). In mollusc tissues, iron is mainly associated to ferritins, whose role includes transport, detoxification and iron storage (Webb et al., 1991). Hemolymph bacterial inhabitants include both mutualistic and pathogenic bacteria that must overcome hemolymph antibacterial activity and compete for the nutrients present in the host sources (Vezzulli et al., 2018).

Although the *V. neptunius* pathogenicity is not yet well known, some studies made with *V. coralliilyticus* could shed some light to the *V. neptunius* virulence factors, since these are two close-related bacteria (Thompson et al., 2003; Sawabe et al., 2013; Dubert et al., 2017). *V. coralliilyticus* pathogenicity depends on the coordinated expression of multiple virulence factors such as flagella, quorum sensing (QS), T6SS, and extracellular products including hemolysins and proteases (Jeffries, 1982; Austin et al., 2005; de O Santos et al., 2011; Kimes et al., 2012; Richards et al., 2015; Guillemette et al., 2020). It has been suggested that siderophore production should play a main role in *Vibrio* infection disease affecting bivalve molluscs (Soto-Rodriguez et al., 2003; Gómez-León et al., 2005; Mechri et al., 2017; Zhang et al., 2020). However, this hypothesis was based on the high frequency of siderophore producer strains found in mollusc microbiota and not in functional analysis (Desriac et al., 2014; Hernández-Robles et al., 2016; Leite et al., 2017).

The present work shows that *V. neptunius* PP-145.98 genome harbours a close homologue of the piscibactin system (*irp* genes), which is part of the highly pathogenicity island *irp*-HPI (Osorio et al., 2006; Balado et al., 2018). This genomic island harbours the genes that encode most functions required for piscibactin biosynthesis (*irp123459*) and uptake (*frpABC*), but lacks a gene homolog to *entD*, which encodes an 4’-phosphopantetheinyl transferase (PPTases) (Osorio et al., 2015; Balado et al., 2018). PPTases are required to activate the non-ribosomal peptide synthetases (NRPSs) assembly lines enabling siderophore synthesis (Orikasa et al., 2006). This function must be supplied by one of the two PPTases found in *V. neptunius* genome (loci WP_206370543.1 and WP_206368753.1). Genetic and chemical data proved that *irp* genes of *V. neptunius* PP-145.98 are expressed and that piscibactin is being synthesized. Since our previous works demonstrated that *V. neptunius* PP-145.98 also produces the siderophore amphibactin (Galvis et al., 2020), *V. neptunius* PP-145.98 would produce two different siderophores, amphibactin and piscibactin, simultaneously. This is not a rare characteristic, since the same feature has been reported in other bacteria such as *Pseudomonas aeruginosa* (Cornelis and Dingemans, 2013) or *Escherichia coli* (Valdebenito et al., 2006), or in the fish pathogens *V. anguillarum* (Balado et al., 2018) and *Aeromonas salmonicida* (Balado et al., 2015). The synthesis of siderophores demands a high energetic cost to the bacterium (Cornelis et al., 2011). Thus, production of redundant siderophore systems by a cell is deleterious (Cordero et al., 2012). However, in some cases, switching between siderophore systems would enhance niche flexibility (Sandy and Butler, 2009; Dumas et al., 2013; Zhang et al., 2019) and pathogenesis (Garcia et al., 2011; Balado et al., 2018).

Inactivation of both siderophore systems, piscibactin and amphibactin, greatly reduced growth capacity of *V. neptunius* under low-iron conditions. Interestingly, amphibactin and piscibactin are siderophores with different chemical characteristics and different affinity for Fe(III). Amphibactin is an amphiphilic siderophore produced by the addition of a variable fatty acid moiety to a head group that coordinates a strong affinity for iron. Thus, amphibactin is mainly associated to the cell membrane, which would minimize the diffusive loss of siderophore in the marine environment (Martinez et al., 2003; Boiteau et al., 2016). On the other hand, piscibactin is a labile yersiniabactin-like siderophore with low affinity for iron (Souto et al., 2012). Congruently, the *V. neptunius* Δ*irp2* single mutant, that only produces amphibactin, showed higher growth under strong iron restriction than the piscibactin-only producer *V. neptunius* Δ*abtF* mutant. The affinity of a siderophore for iron does not predict their impact in virulence, e.g. highly virulent *V. anguillarum* strains produce piscibactin and also vanchrobactin, a siderophore with higher affinity for iron, but piscibactin production is the most relevant for virulence (Balado et al., 2018).

Infection challenges showed that inactivation of siderophore synthesis strongly reduced the mortality caused by *V. neptunius* in clams. Thus, our results proved that siderophore production constitutes a key virulence factor of pathogenic Vibrios in molluscs. Interestingly, inactivation of one of the two siderophore systems (either amphibactin or piscibactin) significantly reduced virulence compared to the wild type strain. This finding showed that the ability to produce both siderophores maximises virulence of *V. neptunius*. In this concern, piscibactin a yersiniabactin-like siderophore, belongs to a type of siderophores that could have other roles during infection besides iron uptake, since they could promote bacterial colonization, dissemination and resistance against phagocytosis (Chaturvedi et al., 2012; Bobrov et al., 2014; Holden and Bachman, 2015; Koh and Henderson, 2015). Thus, piscibactin and amphibactin could have specialized role(s) in the survival of *V. neptunius* within the host and the environment. However, the fact that *V. neptunius* single mutants deficient in each one of these siderophores have showed indistinguishable virulence properties, suggests that both siderophore systems contribute equally to *V. neptunius* virulence.

Although amphibactin is one of the most abundant siderophores in seawater (Boiteau et al., 2016; Gauglitz et al., 2021), transcriptional organization of amphibactin genes was not studied to date. The results demonstrated that amphibactin and piscibactin siderophore systems are significantly expressed when *V. neptunius* is cultivated under low-iron availability *in vitro*. More notably, both siderophore systems are also simultaneously expressed at similar levels when *V. neptunius* is cultivated in bivalve hemolymph (*ex vivo*). The co-expression of siderophores with different chemical properties could contribute to enhance bacterial fitness during host-pathogen interactions (Dumas et al., 2013; Lages et al., 2019). Thus, these results confirm that the high virulence for clams showed by *V. neptunius* wild-type strain must depend on the simultaneous production of piscibactin and amphibactin.

Austin and col. (2005) showed that the *V. neptunius* LMG 20536 and LMG 20610 strains are pathogenic to rainbow trout causing up to 100% mortality at a dose of 10^6^ cells per fish inoculated intraperitoneally. The present work showed that, although PP-145.98 strain harbors the Highly Pathogenicity Island *irp*-HPI and produces the siderophore piscibactin, wild type strain does not cause mortality in turbot (*Scophthalmus maximus*). Interestingly, Austin and col. (2005) also showed that while extracellular products (ECPs) of *V. neptunius* LMG 20610 were highly toxic for fish, ECPs of *V. neptunius* LMG 20536 were almost non toxic (Austin et al., 2005). These findings greatly suggest the existence of a high heterogeneity among *V. neptunius* strains and are in agreement with previous works showing that the pathogenicity mechanisms necessary for infecting fish or shellfish are different (Won and Park, 2008; Zhang et al., 2020).

In a previous work it was found that a high proportion of the *Vibrio*-like isolates from mussel hemolymph were PCR positive for a target in amphibactin outer membrane transporter *abtA* (Galvis et al., 2020). Those *abtA*-positive isolates were mainly non-pathogenic vibrios belonging to the Splendidus clade (Galvis et al., 2020). The *in silico* analysis of 234 genome sequences of well characterized mollusc pathogens belonging to the clades Coralliilyticus, Anguillarum, Harveyi, Orientalis, Pectenecida and Splendidus showed that amphibactin is widespread not only in the Splendidus clade, but also in Orientalis and Coralliilyticus clades. However, *irp*-HPI element encoding piscibactin seems to be limited to the Coralliilyticus clade, since it is present in all *V. neptunius* and *V. ostreicida* genomes and in most *V. coralliilyticus* genomes available. Thus, simultaneous production of both siderophores would be a widespread character in the well-recognized mollusc pathogens *V. coralliilyticus*, *V. neptunius* and *V. ostreicida*. According to the available data, siderophores could have a dual function since they can be key drivers of the microbial community structure and would be key virulence factors that enable infection in an animal host (Vezzulli et al., 2008; Cordero et al., 2012; Kramer et al., 2020; Gauglitz et al., 2021). Rubio-Portillo and col. (2020) showed that siderophore production has a central role in competition among bacteria of mollusc microbiota, enhancing the ability of *Vibrio* coral pathogens such as *V. coralliilyticus* and *V. mediterranei* to invade the host and cause tissue necrosis.

It is known that some bacterial diseases emerged after rapid spread of selectively favoured virulence factors by horizontal gene transfer (HGT) (Bruto et al., 2017; Le Roux and Blokesch, 2018). Molluscs are a major group of marine animals that play key roles in marine ecosystems including water clarification and providing an habitat for other organisms (Schatte Olivier et al., 2020). In addition, molluscs serve as alternative/reservoir hosts for pathogenic bacteria, including human pathogens (Destoumieux-Garzón et al., 2020). Although mollusc microbiota itself would not be a primary source of pathogens for higher animals, the high prevalence of siderophore systems in this niche would imply that the microbiota could serve as test beds (reservoir) of virulence factors like the High-Pathogenicity Island *irp*-HPI encoding the piscibactin system, which is a proved virulence factor in some fish pathogenic bacteria (Osorio et al., 2015; Balado et al., 2018).

In conclusion, synthesis of the siderophores amphibactin and piscibactin, which are widespread in several different *Vibrio* species pathogenic for marine bivalve molluscs, would constitute key virulence factors for disease outbreaks in molluscs aquaculture systems.

## Data Availability Statement

Publicly available datasets were analyzed in this study. This data can be found here: NCBI, accession number: JAFHLB000000000.

## Ethics Statement

The animal study was reviewed and approved by Bioethics Committee of the University of Santiago de Compostela.

## Author Contributions

FG and LA performed the lab experiments. MB, JR, and CJ analyzed the data. FG, LA, CJ, and MB wrote the first draft of the manuscript. MB and ML corrected the draft and built the final version of the manuscript. All authors conceived and designed the study, contributed to manuscript revision, and read and approved the submitted version.

## Funding

This work was supported by grants AGL2017-86183-R (AEI/FEDER, EU), RTI2018-093634-B-C21/C22 (AEI/FEDER, EU) and PID2019-103891RJ-100 (AEI) from the State Agency for Research (AEI) of Spain. AGL2017-86183-R and RTI2018-093634-B-C21/C22 were co-funded by the FEDER Programme from the European Union. Work in University of Santiago de Compostela was also supported by grants GRC2018/018 and 2021-CP112, and in University of A Coruña by grant GRC2018/039 from Xunta de Galicia (Spain). FG was financed with a fellowship ‘Programa de formación de recurso humano de alto nivel doctorado en el exterior’ granted by Colciencias and the government of Norte de Santander, Colombia. LA was financed with a fellowship (ED481A-2019/081) from Xunta de Galicia (Spain), co-financed by ESF (European Social Fund).

## Conflict of Interest

The authors declare that the research was conducted in the absence of any commercial or financial relationships that could be construed as a potential conflict of interest.

## Publisher’s Note

All claims expressed in this article are solely those of the authors and do not necessarily represent those of their affiliated organizations, or those of the publisher, the editors and the reviewers. Any product that may be evaluated in this article, or claim that may be made by its manufacturer, is not guaranteed or endorsed by the publisher.
