# Supplementary material for: Vibrio neptunius Produces Piscibactin and Amphibactin and Both Siderophores Contribute Significantly to Virulence for Clams
Source: Front Cell Infect Microbiol. 2021 Oct 25;11:750567. doi: 10.3389/fcimb.2021.750567 (PMC8573110; doi:10.3389/fcimb.2021.750567)
Supplement: Supplementary file 1 [file Table_1.docx]

**SUPPLEMENTARY INFORMATION**

***Vibrio neptunius* produces piscibactin and amphibactin and both siderophores contribute significantly to virulence for clams**

Fabián Galvis^1^, Lucía Ageitos^2^, Jaime Rodríguez^2^, Carlos Jiménez^2^, Juan L. Barja^1^, Manuel L. Lemos^1^*, Miguel Balado^1^*

^1^Departamento de Microbiología y Parasitología, instituto de Acuicultura y Facultad de Biología-CIBUS, Universidade de Santiago de Compostela, Campus Sur, Santiago de Compostela, Spain.

^2^Centro de Investigacións Científicas Avanzadas (CICA) e Departamento de Química, Facultad de Ciencias, CICA-INIBIC, Universidade da Coruña, A Coruña, Spain.

**Table S1.** Oligonucleotides used for construction of mutants by allelic exchange, transcriptional fusions, and RT-PCR assays.

**Table S2.** Description of piscibactin genes and homology to those found in V. anguillarum and *P. damselae* subsp. *piscicida*.

**Table S3.** Distribution of amphibactin and piscibactin gene systems among mollusc pathogenic *Vibrio* species genomes available in the GeneBanc.

**Table S1.** Oligonucleotides used for construction of mutants by allelic exchange, transcriptional fusions, and RT-PCR assays.

| **Oligonucleotide** | **Sequence**^a^ **(5´→3´)** | | | **Amplification**  **size (bp)** |
| --- | --- | --- | --- | --- |
| ***irp2* defective mutant construction** | | | | |
| irp2 VN XbaI_1* | CGCTCTAGAACTTGCCGCAGGAGGTGGTG | | | 961 |
| irp2 VN BamHI_2 | GGCGGATCCTGACAGCCGTTGCCTCCCAG | | |  |
| irp2 VN BamHI_3 | GGCGGATCCCTGGTCCCAATCTTTGGCCA | | | 980 |
| irp2 VN EcoRI_4* | CGGGAATTCGAGTTGCCCATAAATTCAAT | | |  |
| ***absF* defective mutant construction** | | | |  |
| absF VN XbaI_1* | CGCTCTAGACTGGATGAAATGCCTCTTTC | | | 898 |
| absF VN BamHI_2 | GGCGGATCCATTCGCACCGGCATCTCATG | | |  |
| absF VN BamHI_3 | GGCGGATCCCGTCACAAAACTTTGATCAC | | | 897 |
| absF VN EcoRI_4* | CGGGAATTCCTGGGCCACGCAGAATTTCT | | |  |
| ***abtC* promoter fusion construction** | | | | |
| abtC_XbaI* | GGCTCTAGAAGGAGACGTTGGATATCGAC | | | 705 |
| abtC_BamHI | GGCGGATCCACCATTGTGACCAACAAGAG | | |  |
| ***abtA* promoter fusion construction** | | | | |
| abtA_KpnI* | CGCGGTACCGACTGCTATGTTCCAGGTAC | | | 680 |
| abtA_EcoRI | GCCGAATTCAGACAGAGCGAACGCTACTG | | |  |
| ***absE* promoter fusion construction** | | | | |
| absE_XbaI* | GGCTCTAGAAATGAGCCACAAAGTGACGC | | | 761 |
| absE_BamHI | GGCGGATCCCAACCATAGACGTTGCTGAG | | |  |
| ***araC1* promoter fusion construction** | | | | |
| araC1_XbaI* | CGCTCTAGAGAGGTTGACAGAGTCTCATC | | | 910 |
| araC1_BamHI | CGCGGATCCACACGATGTGGCTTGAGAGC | | |  |
| ***frpA* promoter fusion construction** | |  |  | |
| frpAvn_XbaI* | GGCTCTAGAGATGATGTCCGATGTACACG | | | 740 |
| frpAvn_BamHI | CGGGGATCCGATGATCATCACTTCCTGAC | | |  |
| **LacZ-R*** | CGTAGGTAGTCACGCAACTC | | |  |
| **RT-PCR experiments** | | | | |
| **RT** | TCCGTTGAAACCACGCGAAC | | |  |
| **PCR1** |  | | |  |
| AbtCDB-F | TTTCTGCGACCATATCGTGG | | | 300 |
| AbtCDB-R | AACGCCAAGAGCGATCATGG | | |  |
| **PCR2** |  | | |  |
| AbtE-F | GTGTCACTGCACAAGTTGGG | | | 309 |
| AbtE-R | AGTGATACGAGTCACCAACC | | |  |
| **PCR3** |  | | |  |
| AbsEF-F | ACTTCTTTGCCGCTGCGACG | | | 300 |
| AbsEF-R | TGCACCTCTTCTGCTGGGAC | | |  |
| **PCR4** |  | | |  |
| AbtA/AbsB-F | CGATGCAACAGTTAGTTACC | | | 300 |
| AbtA/AbsB-R | TATCGTTGATGGCACTGCTG | | |  |

*Pimer used to verify mutant construction or *lacZ* fusion by Sanger sequencing

^a^Recognition sequences for restriction enzymes are underlined.

Table S2. Description of piscibactin genes and homology to those found in V. anguillarum and *P. damselae* subsp. *piscicida*.

| **Protein ID in**  ***V. neptunius***  **PP-145.98** | **Predicted function (Gene name)** | **Comparison of identity with *V. neptunius* PP-145.98 with blastn and blastp** | | | | | | | |
| --- | --- | --- | --- | --- | --- | --- | --- | --- | --- |
|  |  | ***V. neptunius* S2394** | | ***V. coralliilyticus* RE22** | | ***V. anguillarum* RV22** | | ***P. damselae* subsp. *piscicida* DI21** | |
|  |  | **DNA** | **AA** | **DNA** | **AA** | **DNA** | **AA** | **DNA** | **AA** |
| [WP_206371146.1](https://www.ncbi.nlm.nih.gov/protein/1997936056) | AraC family transcriptional regulator (*araC1*) | 98 | 99 | 85 | 87 | 71 | 61 | 78 | 82 |
| [WP_045975014.1](https://www.ncbi.nlm.nih.gov/protein/800946767) | AraC family transcriptional regulator  (*araC2*) | 99 | 100 | 84 | 88 | 72 | 63 | 73 | 78 |
| [WP_206371145.1](https://www.ncbi.nlm.nih.gov/protein/1997936055) | TonB-dependent receptor  (*frpA*) | 99 | 99 | 87 | 89 | 66 | 63 | 76 | 80 |
| [WP_206371144.1](https://www.ncbi.nlm.nih.gov/protein/1997936054) | Major facilitator superfamily protein (*irp8*) | 98 | 97 | 82 | 85 | 66 | 60 | 74 | 77 |
| [WP_206371143.1](https://www.ncbi.nlm.nih.gov/protein/1997936053) | Non-ribosomal peptide synthetase (*irp1*) | 99 | 99 | 85 | 87 | 69 | 57 | 72 | 75 |
| [WP_206371142.1](https://www.ncbi.nlm.nih.gov/protein/1997936052) | Non-ribosomal peptide synthetase (*irp2*) | 99 | 99 | 84 | 85 | 72 | 54 | 71 | 71 |
| [WP_206371141.1](https://www.ncbi.nlm.nih.gov/protein/1997936051) | Oxidoreductase involved in siderophore biosynthesis *(irp3)* | 99 | 98 | 85 | 86 | 66 | 59 | 72 | 75 |
| [WP_206371140.1](https://www.ncbi.nlm.nih.gov/protein/1997936050) | Thioesterase protein *(irp4)* | 99 | 98 | 86 | 90 | 68 | 58 | 75 | 75 |
| [WP_206371139.1](https://www.ncbi.nlm.nih.gov/protein/1997936049) | Anthranilate synthase *(irp9)* | 99 | 99 | 84 | 91 | 70 | 61 | 73 | 77 |
| [WP_206371138.1](https://www.ncbi.nlm.nih.gov/protein/1997936048) | 2,3-dihydroxybenzoate-AMP ligase *(irp5)* | 99 | 99 | 82 | 87 | 68 | 65 | 72 | 76 |
| [WP_045975023.1](https://www.ncbi.nlm.nih.gov/protein/800946776) | ABC transporter ATP-binding protein (*mdlB*) | 99 | 100 | 84 | 89 | 67 | 60 | 74 | 80 |
| [WP_206371137.1](https://www.ncbi.nlm.nih.gov/protein/1997936047) | ABC transporter ATP-binding protein (*mdlB*) | 98 | 99 | 84 | 89 | 68 | 59 | 73 | 79 |

Table S3. Distribution of amphibactin and piscibactin gene systems among mollusk pathogenic *Vibrio* species genomes available in the GeneBanc.

| **CLADE/SPECIE/STRAIN** | **PISCIBACTINA** |  | **ANFIBACTINA** |  | **Accession No.** |
| --- | --- | --- | --- | --- | --- |
|  | [**Query Cover**](https://blast.ncbi.nlm.nih.gov/Blast.cgi?CMD=Get&ADV_VIEW=yes&ADV_VIEW=on&ALIGNDB_BATCH_ID=300121191&ALIGNDB_CGI_HOST=blast.be-md.ncbi.nlm.nih.gov&ALIGNDB_CGI_PATH=/ALIGNDB/alndb_asn.cgi&ALIGNDB_MASTER_ALIAS=SD_ALIGNDB_MASTER&ALIGNDB_MAX_ROWS=100&ALIGNDB_ORDER_CLAUSE=seq_evalue%20asc,aln_id%20asc&ALIGNDB_WHERE_CLAUSE=seq_evalue%20is%20not%20null&ALIGNMENTS=100&ALIGNMENT_VIEW=Pairwise&CONFIG_DESCR=2,3,6,7,8,9,10,11,12&DATABASE_SORT=0&DESCRIPTIONS=100&DYNAMIC_FORMAT=on&FIRST_QUERY_NUM=0&FORMAT_NUM_ORG=1&FORMAT_OBJECT=Alignment&FORMAT_PAGE_TARGET=&FORMAT_TYPE=HTML&GET_SEQUENCE=yes&I_THRESH=&LINE_LENGTH=60&MASK_CHAR=2&MASK_COLOR=1&NUM_OVERVIEW=100&PAGE=MegaBlast&QUERY_INDEX=0&QUERY_NUMBER=0&RESULTS_PAGE_TARGET=&RID=5TTUBEAE016&SHOW_LINKOUT=yes&SHOW_OVERVIEW=yes&STEP_NUMBER=&USE_ALIGNDB=true&ADV_VIEW=on&DISPLAY_SORT=4&HSP_SORT=0) **(%)** | [**Per. Ident**](https://blast.ncbi.nlm.nih.gov/Blast.cgi?CMD=Get&ADV_VIEW=yes&ADV_VIEW=on&ALIGNDB_BATCH_ID=300121191&ALIGNDB_CGI_HOST=blast.be-md.ncbi.nlm.nih.gov&ALIGNDB_CGI_PATH=/ALIGNDB/alndb_asn.cgi&ALIGNDB_MASTER_ALIAS=SD_ALIGNDB_MASTER&ALIGNDB_MAX_ROWS=100&ALIGNDB_ORDER_CLAUSE=seq_evalue%20asc,aln_id%20asc&ALIGNDB_WHERE_CLAUSE=seq_evalue%20is%20not%20null&ALIGNMENTS=100&ALIGNMENT_VIEW=Pairwise&CONFIG_DESCR=2,3,6,7,8,9,10,11,12&DATABASE_SORT=0&DESCRIPTIONS=100&DYNAMIC_FORMAT=on&FIRST_QUERY_NUM=0&FORMAT_NUM_ORG=1&FORMAT_OBJECT=Alignment&FORMAT_PAGE_TARGET=&FORMAT_TYPE=HTML&GET_SEQUENCE=yes&I_THRESH=&LINE_LENGTH=60&MASK_CHAR=2&MASK_COLOR=1&NUM_OVERVIEW=100&PAGE=MegaBlast&QUERY_INDEX=0&QUERY_NUMBER=0&RESULTS_PAGE_TARGET=&RID=5TTUBEAE016&SHOW_LINKOUT=yes&SHOW_OVERVIEW=yes&STEP_NUMBER=&USE_ALIGNDB=true&ADV_VIEW=on&DISPLAY_SORT=3&HSP_SORT=3) **(%)** | [**Query Cover**](https://blast.ncbi.nlm.nih.gov/Blast.cgi?CMD=Get&ADV_VIEW=yes&ADV_VIEW=on&ALIGNDB_BATCH_ID=300121191&ALIGNDB_CGI_HOST=blast.be-md.ncbi.nlm.nih.gov&ALIGNDB_CGI_PATH=/ALIGNDB/alndb_asn.cgi&ALIGNDB_MASTER_ALIAS=SD_ALIGNDB_MASTER&ALIGNDB_MAX_ROWS=100&ALIGNDB_ORDER_CLAUSE=seq_evalue%20asc,aln_id%20asc&ALIGNDB_WHERE_CLAUSE=seq_evalue%20is%20not%20null&ALIGNMENTS=100&ALIGNMENT_VIEW=Pairwise&CONFIG_DESCR=2,3,6,7,8,9,10,11,12&DATABASE_SORT=0&DESCRIPTIONS=100&DYNAMIC_FORMAT=on&FIRST_QUERY_NUM=0&FORMAT_NUM_ORG=1&FORMAT_OBJECT=Alignment&FORMAT_PAGE_TARGET=&FORMAT_TYPE=HTML&GET_SEQUENCE=yes&I_THRESH=&LINE_LENGTH=60&MASK_CHAR=2&MASK_COLOR=1&NUM_OVERVIEW=100&PAGE=MegaBlast&QUERY_INDEX=0&QUERY_NUMBER=0&RESULTS_PAGE_TARGET=&RID=5TTUBEAE016&SHOW_LINKOUT=yes&SHOW_OVERVIEW=yes&STEP_NUMBER=&USE_ALIGNDB=true&ADV_VIEW=on&DISPLAY_SORT=4&HSP_SORT=0) **(%)** | [**Per. Ident**](https://blast.ncbi.nlm.nih.gov/Blast.cgi?CMD=Get&ADV_VIEW=yes&ADV_VIEW=on&ALIGNDB_BATCH_ID=300121191&ALIGNDB_CGI_HOST=blast.be-md.ncbi.nlm.nih.gov&ALIGNDB_CGI_PATH=/ALIGNDB/alndb_asn.cgi&ALIGNDB_MASTER_ALIAS=SD_ALIGNDB_MASTER&ALIGNDB_MAX_ROWS=100&ALIGNDB_ORDER_CLAUSE=seq_evalue%20asc,aln_id%20asc&ALIGNDB_WHERE_CLAUSE=seq_evalue%20is%20not%20null&ALIGNMENTS=100&ALIGNMENT_VIEW=Pairwise&CONFIG_DESCR=2,3,6,7,8,9,10,11,12&DATABASE_SORT=0&DESCRIPTIONS=100&DYNAMIC_FORMAT=on&FIRST_QUERY_NUM=0&FORMAT_NUM_ORG=1&FORMAT_OBJECT=Alignment&FORMAT_PAGE_TARGET=&FORMAT_TYPE=HTML&GET_SEQUENCE=yes&I_THRESH=&LINE_LENGTH=60&MASK_CHAR=2&MASK_COLOR=1&NUM_OVERVIEW=100&PAGE=MegaBlast&QUERY_INDEX=0&QUERY_NUMBER=0&RESULTS_PAGE_TARGET=&RID=5TTUBEAE016&SHOW_LINKOUT=yes&SHOW_OVERVIEW=yes&STEP_NUMBER=&USE_ALIGNDB=true&ADV_VIEW=on&DISPLAY_SORT=3&HSP_SORT=3) **(%)** |  |
| **CORALLIILYTICUS**  **CLADE** |  |  |  |  |  |
| ***V. neptunius*** |  |  |  |  |  |
| S2394 | 100 | 94,8 | 99 | 99 | [GCA_000967495.1](https://www.ncbi.nlm.nih.gov/assembly/GCA_000967495.1) |
| PP-256 | 100 | 100 | 100 | 100 | [GCA_017051775.1](https://www.ncbi.nlm.nih.gov/assembly/GCA_017051775.1) |
| PP-259 | 97 | 91 | 99 | 98 | [GCA_017051755.1](https://www.ncbi.nlm.nih.gov/assembly/GCA_017051755.1) |
| PP-307 | 100 | 100 | 100 | 100 | [GCA_017051795.1](https://www.ncbi.nlm.nih.gov/assembly/GCA_017051795.1) |
| PP-313 | 100 | 100 | 100 | 100 | [GCA_017051655.1](https://www.ncbi.nlm.nih.gov/assembly/GCA_017051655.1) |
| ***V. coralliilyticus*** |  |  |  |  |  |
| RE22 | 97 | 84,6 | 96 | 92,4 | [[GCA_001297935.1](https://www.ncbi.nlm.nih.gov/assembly/GCA_001297935.1)](https://blast.ncbi.nlm.nih.gov/Blast.cgi#alnHdr_930107324) |
| S2043 | 97 | 84,6 | 92 | 93,2 | [[GCA_000967485.1](https://www.ncbi.nlm.nih.gov/assembly/GCA_000967485.1)](https://blast.ncbi.nlm.nih.gov/Blast.cgi#alnHdr_798719221) |
| CN26H-1 | 97 | 84,5 | 94 | 92,7 | [GCA_013266555.1](https://www.ncbi.nlm.nih.gov/assembly/GCA_013266555.1) |
| RE87 | 97 | 84,5 | 95 | 92,8 | [[GCA_002286655.1](https://www.ncbi.nlm.nih.gov/assembly/GCA_002286655.1)](https://blast.ncbi.nlm.nih.gov/Blast.cgi#alnHdr_1239992497) |
| P1 | 97 | 84,5 | 94 | 93,1 | [[GCA_000195475.2](https://www.ncbi.nlm.nih.gov/assembly/GCA_000195475.2)](https://blast.ncbi.nlm.nih.gov/Blast.cgi#alnHdr_328565694) |
| ATCC BAA-450 | 97 | 84,5 | 96 | 91,7 | [[GCA_000176135.1](https://www.ncbi.nlm.nih.gov/assembly/GCA_000176135.1)](https://blast.ncbi.nlm.nih.gov/Blast.cgi#alnHdr_260605565) |
| CN52H-1 | 97 | 84,5 | 94 | 93,3 | [GCA_013266605.1](https://www.ncbi.nlm.nih.gov/assembly/GCA_013266605.1) |
| NA0301 | 97 | 84,5 | 95 | 93,1 | [[GCA_002742585.1](https://www.ncbi.nlm.nih.gov/assembly/GCA_002742585.1),](https://blast.ncbi.nlm.nih.gov/Blast.cgi#alnHdr_1273326397) |
| 09-121-3 | 97 | 84,5 | 99 | 92,2 | [[GCA_013114385.1](https://www.ncbi.nlm.nih.gov/assembly/GCA_013114385.1)](https://blast.ncbi.nlm.nih.gov/Blast.cgi#alnHdr_VTXP01000001) |
| C154 | 97 | 84,5 | 93 | 92,6 | [[GCA_013113905.1](https://www.ncbi.nlm.nih.gov/assembly/GCA_013113905.1)](https://blast.ncbi.nlm.nih.gov/Blast.cgi#alnHdr_VTYL01000001) |
| 071316B | 97 | 84,5 | 93 | 92,6 | [[GCA_013114065.1](https://www.ncbi.nlm.nih.gov/assembly/GCA_013114065.1)](https://blast.ncbi.nlm.nih.gov/Blast.cgi#alnHdr_VTYG01000001) |
| OfT6-17 | 97 | 84,4 | 93 | 92,5 | [GCA_013266655.1](https://www.ncbi.nlm.nih.gov/assembly/GCA_013266655.1) |
| OfT6-21 | 97 | 84,4 | 93 | 92,5 | [GCA_013266665.1](https://www.ncbi.nlm.nih.gov/assembly/GCA_013266665.1) |
| OfT7-21 | 97 | 84,4 | 90 | 92,5 | [[GCA_013266615.1](https://www.ncbi.nlm.nih.gov/assembly/GCA_013266615.1)](https://blast.ncbi.nlm.nih.gov/Blast.cgi#alnHdr_JABSMY010000043) |
| RE90 | 97 | 84,4 | 93 | 92,3 | [[GCA_013113815.1](https://www.ncbi.nlm.nih.gov/assembly/GCA_013113815.1)](https://blast.ncbi.nlm.nih.gov/Blast.cgi#alnHdr_VTYR01000007) |
| OCN008 | 97 | 85,3 | 97 | 92 | [[GCA_000461895.1](https://www.ncbi.nlm.nih.gov/assembly/GCA_000461895.1)](https://blast.ncbi.nlm.nih.gov/Blast.cgi#alnHdr_536748777) |
| 081216C | - | - | 100 | 92 | [GCA_013114055.1](https://www.ncbi.nlm.nih.gov/assembly/GCA_013114055.1) |
| 080116A | - | - | 100 | 91,9 | [GCA_002286405.1](https://www.ncbi.nlm.nih.gov/assembly/GCA_002286405.1) |
| MCA-25 | - | - | 100 | 91,9 | [GCA_013266565.1](https://www.ncbi.nlm.nih.gov/assembly/GCA_013266565.1) |
| MCA-32 | - | - | 100 | 91,9 | [GCA_013266595.1](https://www.ncbi.nlm.nih.gov/assembly/GCA_013266595.1) |
| AIC-5 | - | - | 99 | 92,3 | [GCA_013114665.1](https://www.ncbi.nlm.nih.gov/assembly/GCA_013114665.1) |
| AIC-7 | - | - | 99 | 92,3 | [GCA_002287625.1](https://www.ncbi.nlm.nih.gov/assembly/GCA_002287625.1) |
| Vic-OC-068 | - | - | 99 | 92,2 | [GCA_013364385.1](https://www.ncbi.nlm.nih.gov/assembly/GCA_013364385.1) |
| ATCC 19105 | - | - | 98 | 92,1 | [GCA_013114685.1](https://www.ncbi.nlm.nih.gov/assembly/GCA_013114685.1) |
| X00-12-4 | - | - | 95 | 92,9 | [GCA_013113805.1](https://www.ncbi.nlm.nih.gov/assembly/GCA_013113805.1) |
| 58 | - | - | 99 | 92,3 | [GCA_001693615.1](https://www.ncbi.nlm.nih.gov/assembly/GCA_001693615.1) |
| OCN014 | 97 | 84,9 | 94 | 92,7 | [GCA_000763535.2](https://www.ncbi.nlm.nih.gov/assembly/GCA_000763535.2) |
| SNUTY-1 | 97 | 84 | 94 | 93 | [GCA_002073995.1](https://www.ncbi.nlm.nih.gov/assembly/GCA_002073995.1) |
| RE98 | - | - | 95 | 93 | [GCA_000772065.1](https://www.ncbi.nlm.nih.gov/assembly/GCA_000772065.1) |
| ***V. ostreicida*** |  |  |  |  |  |
| 203 | 57 | 67,1 | 86 | 66,8 | [GCA_001957165.1](https://www.ncbi.nlm.nih.gov/assembly/GCA_001957165.1) |
| UCD-KL16 | 58 | 67 | 86 | 66,7 | [GCA_013074385.2](https://www.ncbi.nlm.nih.gov/assembly/GCA_013074385.2) |
| **PECTENICIDA**  **CLADE** |  |  |  |  |  |
| ***V. pectenicida*** |  |  |  |  |  |
| CAIM 594 | - | - | - | - | [GCA_003937805.1](https://www.ncbi.nlm.nih.gov/assembly/GCA_003937805.1) |
| 99-46-Y | - | - | - | - | [GCA_013114615.1](https://www.ncbi.nlm.nih.gov/assembly/GCA_013114615.1) |
| **ORIENTALIS**  **CLADE** |  |  |  |  |  |
| ***V. bivalvicida*** |  |  |  |  |  |
| 605 | - | - | 97 | 87,5 | [GCA_001399455.2](https://www.ncbi.nlm.nih.gov/assembly/GCF_001399455.2/) |
| ***V. europaeus*** |  |  |  |  |  |
| PP-638 | - | - | 95 | 87 | [GCA_001695575.1](https://www.ncbi.nlm.nih.gov/assembly/GCA_001695575.1) |
| 071316F | - | - | 95 | 87 | [GCA_013114045.1](https://www.ncbi.nlm.nih.gov/assembly/GCA_013114045.1) |
| NPI-1 | - | - | - | - | [GCA_013154935.1](https://www.ncbi.nlm.nih.gov/assembly/GCA_013154935.1) |
| 07/118 T2 | - | - | - | - | [GCA_015654285.1](https://www.ncbi.nlm.nih.gov/assembly/GCA_015654285.1) |
| ***V. tubiashii*** |  |  |  |  |  |
| 01-65-5-1 | - | - | 95 | 88,4 | [GCA_013114395.1](https://www.ncbi.nlm.nih.gov/assembly/GCA_013114395.1) |
| ATCC 19109 | - | - | 56 | 88 | [GCA_000772105.1](https://www.ncbi.nlm.nih.gov/assembly/GCA_000772105.1) |
| NCIMB 1337 | - | - | 95 | 88 | [GCA_000259295.1](https://www.ncbi.nlm.nih.gov/assembly/GCA_000259295.1) |
| **HARVEYI**  **CLADE** |  |  |  |  |  |
| ***V. alginolyiticus*** |  |  |  |  |  |
| NBRC 15630 | - | - | - | - | [GCA_000467145.1](https://www.ncbi.nlm.nih.gov/assembly/GCA_000467145.1) |
| 138-2 | - | - | - | - | [GCA_012151155.1](https://www.ncbi.nlm.nih.gov/assembly/GCA_012151155.1) |
| YM19 | - | - | - | - | [GCA_012151455.1](https://www.ncbi.nlm.nih.gov/assembly/GCA_012151455.1) |
| BNVF9 | - | - | - | - | [GCA_903986595.1](https://www.ncbi.nlm.nih.gov/assembly/GCA_903986595.1) |
| NCTC12160 | - | - | - | - | [GCA_900460285.1](https://www.ncbi.nlm.nih.gov/assembly/GCA_900460285.1) |
| 12G01 | - | - | - | - | [GCA_000153505.1](https://www.ncbi.nlm.nih.gov/assembly/GCA_000153505.1) |
| ATCC 17749 | - | - | - | - | [GCA_000354175.2](https://www.ncbi.nlm.nih.gov/assembly/GCA_000354175.2) |
| FDAARGOS_98 | - | - | - | - | [GCA_001525595.2](https://www.ncbi.nlm.nih.gov/assembly/GCA_001525595.2) |
| EPGS | - | - | - | - | [GCA_001273715.1](https://www.ncbi.nlm.nih.gov/assembly/GCA_001273715.1) |
| 2014V-1011 | - | - | - | - | [GCA_009763085.1](https://www.ncbi.nlm.nih.gov/assembly/GCA_009763085.1) |
| BSW8 | - | - | - | - | [GCA_000834155.1](https://www.ncbi.nlm.nih.gov/assembly/GCA_000834155.1) |
| BSW15 | - | - | - | - | [GCA_000834085.1](https://www.ncbi.nlm.nih.gov/assembly/GCA_000834085.1) |
| VN-7501 | - | - | - | - | [GCA_001617475.1](https://www.ncbi.nlm.nih.gov/assembly/GCA_001617475.1) |
| V1 | - | - | - | - | [GCA_001013315.1](https://www.ncbi.nlm.nih.gov/assembly/GCA_001013315.1) |
| RM-10-2 | - | - | - | - | [GCA_001267615.1](https://www.ncbi.nlm.nih.gov/assembly/GCA_001267615.1) |
| UCD-30C | - | - | - | - | [GCA_001306785.1](https://www.ncbi.nlm.nih.gov/assembly/GCA_001306785.1) |
| UCD-32C | - | - | - | - | [GCA_001306825.1](https://www.ncbi.nlm.nih.gov/assembly/GCA_001306825.1) |
| UCD-53C | - | - | - | - | [GCA_001306845.1](https://www.ncbi.nlm.nih.gov/assembly/GCA_001306845.1) |
| UCD-9C | - | - | - | - | [GCA_001306875.1](https://www.ncbi.nlm.nih.gov/assembly/GCA_001306875.1) |
| ATCC 33787 | - | - | - | - | [GCA_001469735.1](https://www.ncbi.nlm.nih.gov/assembly/GCA_001469735.1) |
| ANC4-19 | - | - | - | - | [GCA_001584265.1](https://www.ncbi.nlm.nih.gov/assembly/GCA_001584265.1) |
| ZJ-T | - | - | - | - | [GCA_001679745.1](https://www.ncbi.nlm.nih.gov/assembly/GCA_001679745.1) |
| 10N.261.52.A3 | - | - | - | - | [GCA_005145885.1](https://www.ncbi.nlm.nih.gov/assembly/GCA_005145885.1) |
| K01M1 | - | - | - | - | [GCA_002119505.2](https://www.ncbi.nlm.nih.gov/assembly/GCA_002119505.2) |
| K04M1 | - | - | - | - | [GCA_003613035.1](https://www.ncbi.nlm.nih.gov/assembly/GCA_003613035.1) |
| K04M3 | - | - | - | - | [GCA_002149105.1](https://www.ncbi.nlm.nih.gov/assembly/GCA_002149105.1) |
| K04M5 | - | - | - | - | [GCA_002119545.1](https://www.ncbi.nlm.nih.gov/assembly/GCA_002119545.1) |
| K05K4 | - | - | - | - | [GCA_003613065.1](https://www.ncbi.nlm.nih.gov/assembly/GCA_003613065.1) |
| K06K5 | - | - | - | - | [GCA_002119565.1](https://www.ncbi.nlm.nih.gov/assembly/GCA_002119565.1) |
| K08M3 | - | - | - | - | [GCA_002149085.1](https://www.ncbi.nlm.nih.gov/assembly/GCA_002149085.1) |
| K08M4 | - | - | - | - | [GCA_002119525.1](https://www.ncbi.nlm.nih.gov/assembly/GCA_002119525.1) |
| K09K1 | - | - | - | - | [GCA_002149065.1](https://www.ncbi.nlm.nih.gov/assembly/GCA_002149065.1) |
| K10K4 | - | - | - | - | [GCA_002119585.1](https://www.ncbi.nlm.nih.gov/assembly/GCA_002119585.1) |
| QD-5 | - | - | - | - | [GCA_002114195.1](https://www.ncbi.nlm.nih.gov/assembly/GCA_002114195.1) |
| BM517 | - | - | - | - | [GCA_002868815.1](https://www.ncbi.nlm.nih.gov/assembly/GCA_002868815.1) |
| 40B | - | - | - | - | [GCA_000176055.1](https://www.ncbi.nlm.nih.gov/assembly/GCA_000176055.1) |
| Hep-1a-2 | - | - | - | - | [GCA_006124875.1](https://www.ncbi.nlm.nih.gov/assembly/GCA_006124875.1) |
| LF TCBS 15 | - | - | - | - | [GCA_004283075.1](https://www.ncbi.nlm.nih.gov/assembly/GCA_004283075.1) |
| DFP1994 | - | - | - | - | [GCA_014769135.1](https://www.ncbi.nlm.nih.gov/assembly/GCA_014769135.1) |
| M3-10 | - | - | - | - | [GCA_008271565.1](https://www.ncbi.nlm.nih.gov/assembly/GCA_008271565.1) |
| BR233 | - | - | - | - | [GCA_004349375.1](https://www.ncbi.nlm.nih.gov/assembly/GCA_004349375.1) |
| FA2 | - | - | - | - | [GCA_011801435.1](https://www.ncbi.nlm.nih.gov/assembly/GCA_011801435.1) |
| 062916C | - | - | - | - | [GCA_013114005.1](https://www.ncbi.nlm.nih.gov/assembly/GCA_013114005.1) |
| S6-61 | - | - | - | - | [GCA_008921745.1](https://www.ncbi.nlm.nih.gov/assembly/GCA_008921745.1) |
| Vb1167 | - | - | - | - | [GCA_012956205.1](https://www.ncbi.nlm.nih.gov/assembly/GCA_012956205.1) |
| GS_MYPK1 | - | - | - | - | [GCA_013368735.1](https://www.ncbi.nlm.nih.gov/assembly/GCA_013368735.1) |
| Vb1833 | - | - | - | - | [GCA_014274185.1](https://www.ncbi.nlm.nih.gov/assembly/GCA_014274185.1) |
| VP318 | - | - | - | - | [GCA_016823165.1](https://www.ncbi.nlm.nih.gov/assembly/GCA_016823165.1) |
| AP-1 | - | - | - | - | [GCA_016937625.1](https://www.ncbi.nlm.nih.gov/assembly/GCA_016937625.1) |
| E06333 | - | - | - | - | [GCA_017161465.1](https://www.ncbi.nlm.nih.gov/assembly/GCA_017161465.1) |
| Vb2145 | - | - | - | - | [GCA_017313625.1](https://www.ncbi.nlm.nih.gov/assembly/GCA_017313625.1) |
| BW13 | - | - | - | - | [GCA_018831165.1](https://www.ncbi.nlm.nih.gov/assembly/GCA_018831165.1) |
| VA114 | - | - | - | - | [GCA_018458125.1](https://www.ncbi.nlm.nih.gov/assembly/GCA_018458125.1) |
| VaAres1_A | - | - | - | - | [GCA_018779875.1](https://www.ncbi.nlm.nih.gov/assembly/GCA_018779875.1) |
| **ANGUILLARUM**  **CLADE** |  |  |  |  |  |
| ***V. aestuarianus*** |  |  |  |  |  |
| O-00-16-10 | - | - | - | - | [GCA_013114495.1](https://www.ncbi.nlm.nih.gov/assembly/GCA_013114495.1) |
| 03/008t | - | - | - | - | [GCA_011090235.1](https://www.ncbi.nlm.nih.gov/assembly/GCA_011090235.1) |
| 12/122 3T3 | - | - | - | - | [GCA_011090275.1](https://www.ncbi.nlm.nih.gov/assembly/GCA_011090275.1) |
| 15/075 3T2 | - | - | - | - | [GCA_011090175.1](https://www.ncbi.nlm.nih.gov/assembly/GCA_011090175.1) |
| 15/064 3T2 | - | - | - | - | [GCA_011090265.1](https://www.ncbi.nlm.nih.gov/assembly/GCA_011090265.1) |
| 15/061_1T1 | - | - | - | - | [GCA_011090225.1](https://www.ncbi.nlm.nih.gov/assembly/GCA_011090225.1) |
| 02/041 | - | - | - | - | [GCA_012395215.1](https://www.ncbi.nlm.nih.gov/assembly/GCA_012395215.1) |
| LMG 7909 | - | - | - | - | [GCA_012395185.1](https://www.ncbi.nlm.nih.gov/assembly/GCA_012395185.1) |
| 01/151 | - | - | - | - | [GCA_012689365.1](https://www.ncbi.nlm.nih.gov/assembly/GCA_012689365.1) |
| 07/115 | - | - | - | - | [GCA_012689305.1](https://www.ncbi.nlm.nih.gov/assembly/GCA_012689305.1) |
| 01/032 | - | - | - | - | [GCA_012689315.1](https://www.ncbi.nlm.nih.gov/assembly/GCA_012689315.1) |
| 01/308 | - | - | - | - | [GCA_012689405.1](https://www.ncbi.nlm.nih.gov/assembly/GCA_012689405.1) |
| **SPLENDIDUS**  **CLADE** |  |  |  |  |  |
| ***V. splendidus*** |  |  |  |  |  |
| ZS-139 | - | - | 57 | 71 | [GCA_000272225.2](https://www.ncbi.nlm.nih.gov/assembly/GCA_000272225.2) |
| ZF-90 | - | - | 62 | 70,6 | [GCA_000272125.2](https://www.ncbi.nlm.nih.gov/assembly/GCA_000272125.2) |
| 10N.222.51.F9 | - | - | 60 | 70,35 | [GCA_002875395.1](https://www.ncbi.nlm.nih.gov/assembly/GCA_002875395.1) |
| 10N.222.52.F3 | - | - | 58 | 70,3 | [GCA_002876645.1](https://www.ncbi.nlm.nih.gov/assembly/GCA_002876645.1) |
| 10N.286.45.F12 | - | - | 58 | 70,8 | [GCA_002872895.1](https://www.ncbi.nlm.nih.gov/assembly/GCA_002872895.1) |
| 10N.261.49.A1 | - | - | 59 | 70,4 | [GCA_002876905.1](https://www.ncbi.nlm.nih.gov/assembly/GCA_002876905.1) |
| 10N.261.45.E8 | - | - | 59 | 70,4 | [GCA_002876785.1](https://www.ncbi.nlm.nih.gov/assembly/GCA_002876785.1) |
| 10N.286.46.A4 | - | - | 58 | 70,4 | [GCA_002874245.1](https://www.ncbi.nlm.nih.gov/assembly/GCA_002874245.1) |
| 1A01 | - | - | 60 | 70,8 | [GCA_002700025.1](https://www.ncbi.nlm.nih.gov/assembly/GCA_002700025.1) |
| ZS_173 | - | - | 62 | 70,6 | [GCA_003050225.1](https://www.ncbi.nlm.nih.gov/assembly/GCA_003050225.1) |
| CECT 8714 | - | - | - | - | [GCA_900089845.1](https://www.ncbi.nlm.nih.gov/assembly/GCA_900089845.1) |
| 12B01 | - | - | - | - | [GCA_000152765.1](https://www.ncbi.nlm.nih.gov/assembly/GCA_000152765.1) |
| 12E03 | - | - | - | - | [GCA_000272105.2](https://www.ncbi.nlm.nih.gov/assembly/GCA_000272105.2) |
| 5S-101 | - | - | - | - | [GCA_000272245.2](https://www.ncbi.nlm.nih.gov/assembly/GCA_000272245.2) |
| FF-500 | - | - | - | - | [GCA_000272265.2](https://www.ncbi.nlm.nih.gov/assembly/GCA_000272265.2) |
| FF-6 | - | - | - | - | [GCA_000272325.2](https://www.ncbi.nlm.nih.gov/assembly/GCA_000272325.2) |
| 1F-157 | - | - | - | - | [GCA_000272345.2](https://www.ncbi.nlm.nih.gov/assembly/GCA_000272345.2) |
| 0407ZC148 | - | - | - | - | [GCA_000272285.1](https://www.ncbi.nlm.nih.gov/assembly/GCA_000272285.1) |
| 1S-124 | - | - | - | - | [GCA_000272305.2](https://www.ncbi.nlm.nih.gov/assembly/GCA_000272305.2) |
| LRBAC | - | - | - | - | [GCA_003732565.1](https://www.ncbi.nlm.nih.gov/assembly/GCA_003732565.1) |
| UCD-SED7 | - | - | - | - | [GCA_001306195.1](https://www.ncbi.nlm.nih.gov/assembly/GCA_001306195.1) |
| UCD-SED10 | - | - | - | - | [GCA_001306215.1](https://www.ncbi.nlm.nih.gov/assembly/GCA_001306215.1) |
| MARa | - | - | - | - | [GCA_001558055.1](https://www.ncbi.nlm.nih.gov/assembly/GCA_001558055.1) |
| 13B01 | - | - | - | - | [GCA_001691275.1](https://www.ncbi.nlm.nih.gov/assembly/GCA_001691275.1) |
| UCD-FRSSP16_15 | - | - | - | - | [GCA_001676015.1](https://www.ncbi.nlm.nih.gov/assembly/GCA_001676015.1) |
| 10N.286.52.F10 | - | - | - | - | [GCA_002873735.1](https://www.ncbi.nlm.nih.gov/assembly/GCA_002873735.1) |
| CAIM 1923 | - | - | - | - | [GCA_003573805.1](https://www.ncbi.nlm.nih.gov/assembly/GCA_003573805.1) |
| VaAn | - | - | - | - | [GCA_002078155.1](https://www.ncbi.nlm.nih.gov/assembly/GCA_002078155.1) |
| ORI231 | - | - | - | - | [GCA_002954555.1](https://www.ncbi.nlm.nih.gov/assembly/GCA_002954555.1) |
| OU02 | - | - | - | - | [GCA_003408655.1](https://www.ncbi.nlm.nih.gov/assembly/GCA_003408655.1) |
| DSM 19640 | - | - | - | - | [GCA_002737025.1](https://www.ncbi.nlm.nih.gov/assembly/GCA_002737025.1) |
| 3C02 | - | - | - | - | [GCA_018729395.1](https://www.ncbi.nlm.nih.gov/assembly/GCA_018729395.1) |
| 5S_210 | - | - | - | - | [GCA_003050445.1](https://www.ncbi.nlm.nih.gov/assembly/GCA_003050445.1) |
| G2R10 | - | - | - | - | [GCA_018860405.1](https://www.ncbi.nlm.nih.gov/assembly/GCA_018860405.1) |
| BST398 | - | - | - | - | [GCA_003345295.1](https://www.ncbi.nlm.nih.gov/assembly/GCA_003345295.1) |
| S7 | - | - | - | - | [GCA_007858915.1](https://www.ncbi.nlm.nih.gov/assembly/GCA_007858915.1) |
| 99-70-6B3 | - | - | - | - | [GCA_013114435.1](https://www.ncbi.nlm.nih.gov/assembly/GCA_013114435.1) |
| 07-146-1 | - | - | - | - | [GCA_013114485.1](https://www.ncbi.nlm.nih.gov/assembly/GCA_013114485.1) |
| 99-70-13A3 | - | - | - | - | [GCA_013114445.1](https://www.ncbi.nlm.nih.gov/assembly/GCA_013114445.1) |
| X00-12-3 | - | - | - | - | [GCA_013114425.1](https://www.ncbi.nlm.nih.gov/assembly/GCA_013114425.1) |
| ATCC 33789 | - | - | - | - | [GCA_000222625.2](https://www.ncbi.nlm.nih.gov/assembly/GCA_000222625.2) |
| 12F01 | - | - | - | - | [GCA_000256485.1](https://www.ncbi.nlm.nih.gov/assembly/GCA_000256485.1) |
| 10N.261.46.E9 | - | - | - | - | [GCA_002876825.1](https://www.ncbi.nlm.nih.gov/assembly/GCA_002876825.1) |
| NCCB 53037 | - | - | - | - | [GCA_001558015.1](https://www.ncbi.nlm.nih.gov/assembly/GCA_001558015.1) |
| MOR2 | - | - | - | - | [GCA_001557765.1](https://www.ncbi.nlm.nih.gov/assembly/GCA_001557765.1) |
| MOR1 | - | - | - | - | [GCA_001557875.1](https://www.ncbi.nlm.nih.gov/assembly/GCA_001557875.1) |
| 10N.286.49.F1 | - | - | - | - | [GCA_002873455.1](https://www.ncbi.nlm.nih.gov/assembly/GCA_002873455.1) |
| 10N.222.51.F8 | - | - | - | - | [GCA_002875345.1](https://www.ncbi.nlm.nih.gov/assembly/GCA_002875345.1) |
| 10N.222.54.F7 | - | - | - | - | [GCA_002875495.1](https://www.ncbi.nlm.nih.gov/assembly/GCA_002875495.1) |
| 10N.222.52.A7 | - | - | - | - | [GCA_002876565.1](https://www.ncbi.nlm.nih.gov/assembly/GCA_002876565.1) |
| 10N.261.45.A2 | - | - | - | - | [GCA_002875585.1](https://www.ncbi.nlm.nih.gov/assembly/GCA_002875585.1) |
| 10N.261.52.F2 | - | - | - | - | [GCA_002877195.1](https://www.ncbi.nlm.nih.gov/assembly/GCA_002877195.1) |
| 10N.261.48.B7 | - | - | - | - | [GCA_002876045.1](https://www.ncbi.nlm.nih.gov/assembly/GCA_002876045.1) |
| 10N.286.45.A10 | - | - | - | - | [GCA_002877525.1](https://www.ncbi.nlm.nih.gov/assembly/GCA_002877525.1) |
| 10N.286.48.F4 | - | - | - | - | [GCA_002878565.1](https://www.ncbi.nlm.nih.gov/assembly/GCA_002878565.1) |
| ZS_90 | - | - | - | - | [GCA_003050125.1](https://www.ncbi.nlm.nih.gov/assembly/GCA_003050125.1) |
| 1S_14 | - | - | - | - | [GCA_003050485.1](https://www.ncbi.nlm.nih.gov/assembly/GCA_003050485.1) |
| 1S_296 | - | - | - | - | [GCA_003050465.1](https://www.ncbi.nlm.nih.gov/assembly/GCA_003050465.1) |
| 5S_57 | - | - | - | - | [GCA_003050255.1](https://www.ncbi.nlm.nih.gov/assembly/GCA_003050255.1) |
| 5S_226 | - | - | - | - | [GCA_003050345.1](https://www.ncbi.nlm.nih.gov/assembly/GCA_003050345.1) |
| ZS_82 | - | - | - | - | [GCA_003050175.1](https://www.ncbi.nlm.nih.gov/assembly/GCA_003050175.1) |
| 5S_245 | - | - | - | - | [GCA_003050385.1](https://www.ncbi.nlm.nih.gov/assembly/GCA_003050385.1) |
| ZS_107 | - | - | - | - | [GCA_003049945.1](https://www.ncbi.nlm.nih.gov/assembly/GCA_003049945.1) |
| 5S_238 | - | - | - | - | [GCA_003050425.1](https://www.ncbi.nlm.nih.gov/assembly/GCA_003050425.1) |
| ZS_181 | - | - | - | - | [GCA_003050245.1](https://www.ncbi.nlm.nih.gov/assembly/GCA_003050245.1) |
| 5S_279 | - | - | - | - | [GCA_003050355.1](https://www.ncbi.nlm.nih.gov/assembly/GCA_003050355.1) |
| 1S_113 | - | - | - | - | [GCA_003050505.1](https://www.ncbi.nlm.nih.gov/assembly/GCA_003050505.1) |
| 5S_283 | - | - | - | - | [GCA_003050285.1](https://www.ncbi.nlm.nih.gov/assembly/GCA_003050285.1) |
| 1S_146 | - | - | - | - | [GCA_003050545.1](https://www.ncbi.nlm.nih.gov/assembly/GCA_003050545.1) |
| ZS_58 | - | - | - | - | [GCA_003049845.1](https://www.ncbi.nlm.nih.gov/assembly/GCA_003049845.1) |
| ZF_41 | - | - | - | - | [GCA_003049865.1](https://www.ncbi.nlm.nih.gov/assembly/GCA_003049865.1) |
| 5S_122 | - | - | - | - | [GCA_003050405.1](https://www.ncbi.nlm.nih.gov/assembly/GCA_003050405.1) |
| ZS_198 | - | - | - | - | [GCA_003050205.1](https://www.ncbi.nlm.nih.gov/assembly/GCA_003050205.1) |
| 1S_129 | - | - | - | - | [GCA_003050025.1](https://www.ncbi.nlm.nih.gov/assembly/GCA_003050025.1) |
| ZS_2 | - | - | - | - | [GCA_003050165.1](https://www.ncbi.nlm.nih.gov/assembly/GCA_003050165.1) |
| ZS_117 | - | - | - | - | [GCA_003050005.1](https://www.ncbi.nlm.nih.gov/assembly/GCA_003050005.1) |
| ZS_138 | - | - | - | - | [GCA_003049975.1](https://www.ncbi.nlm.nih.gov/assembly/GCA_003049975.1) |
| ZS_213 | - | - | - | - | [GCA_003049925.1](https://www.ncbi.nlm.nih.gov/assembly/GCA_003049925.1) |
| ZS_185 | - | - | - | - | [GCA_003049855.1](https://www.ncbi.nlm.nih.gov/assembly/GCA_003049855.1) |
| 1F_55 | - | - | - | - | [GCA_003050045.1](https://www.ncbi.nlm.nih.gov/assembly/GCA_003050045.1) |
| ***V. tasmaniensis*** |  |  |  |  |  |
| UCD-FRSSP16_25 | - | - | 58 | 71 | [GCA_001675865.1](https://www.ncbi.nlm.nih.gov/assembly/GCA_001675865.1) |
| UCD-FRSSP16_35 | - | - | 56 | 71 | [GCA_001675955.1](https://www.ncbi.nlm.nih.gov/assembly/GCA_001675955.1) |
| ZS-17 | - | - | 58 | 70 | [GCA_000272445.2](https://www.ncbi.nlm.nih.gov/assembly/GCA_000272445.2) |
| SM1924 | - | - | 57 | 71 | [GCA_007786305.1](https://www.ncbi.nlm.nih.gov/assembly/GCA_007786305.1) |
| 1F-187 | - | - | - | - | [GCA_000272405.2](https://www.ncbi.nlm.nih.gov/assembly/GCA_000272405.2) |
| 1F-155 | - | - | - | - | [GCA_000272385.2](https://www.ncbi.nlm.nih.gov/assembly/GCA_000272385.2) |
| 1F-267 | - | - | - | - | [GCA_000272365.2](https://www.ncbi.nlm.nih.gov/assembly/GCA_000272365.2) |
| 5F-79 | - | - | - | - | [GCA_000272425.1](https://www.ncbi.nlm.nih.gov/assembly/GCA_000272425.1) |
| 10N.222.45.E7 | - | - | - | - | [GCA_005146675.1](https://www.ncbi.nlm.nih.gov/assembly/GCA_005146675.1) |
| 10N.222.48.A2 | - | - | - | - | [GCA_002875145.1](https://www.ncbi.nlm.nih.gov/assembly/GCA_002875145.1) |
| 10N.222.45.A8 | - | - | - | - | [GCA_005146825.1](https://www.ncbi.nlm.nih.gov/assembly/GCA_005146825.1) |
| 10N.222.45.A2 | - | - | - | - | [GCA_005146875.1](https://www.ncbi.nlm.nih.gov/assembly/GCA_005146875.1) |
| 10N.222.45.A1 | - | - | - | - | [GCA_005146635.1](https://www.ncbi.nlm.nih.gov/assembly/GCA_005146635.1) |
| 10N.222.45.A4 | - | - | - | - | [GCA_005146895.1](https://www.ncbi.nlm.nih.gov/assembly/GCA_005146895.1) |
| 10N.261.51.E11 | - | - | - | - | [GCA_002876245.1](https://www.ncbi.nlm.nih.gov/assembly/GCA_002876245.1) |
| 10N.261.52.A6 | - | - | - | - | [GCA_002876335.1](https://www.ncbi.nlm.nih.gov/assembly/GCA_002876335.1) |
| 10N.222.45.A3 | - | - | - | - | [GCA_005146915.1](https://www.ncbi.nlm.nih.gov/assembly/GCA_005146915.1) |
| 10N.222.51.A7 | - | - | - | - | [GCA_002876485.1](https://www.ncbi.nlm.nih.gov/assembly/GCA_002876485.1) |
| 10N.222.45.A5 | - | - | - | - | [GCA_005146845.1](https://www.ncbi.nlm.nih.gov/assembly/GCA_005146845.1) |
| J5-9 | - | - | - | - | [GCA_004761895.1](https://www.ncbi.nlm.nih.gov/assembly/GCA_004761895.1) |
| J5-13 | - | - | - | - | [GCA_004764445.1](https://www.ncbi.nlm.nih.gov/assembly/GCA_004764445.1) |
| J0-13 | - | - | - | - | [GCA_004761905.1](https://www.ncbi.nlm.nih.gov/assembly/GCA_004761905.1) |
| LMG 20012 | - | - | - | - | [GCA_006333845.1](https://www.ncbi.nlm.nih.gov/assembly/GCA_006333845.1) |
| ***V. crassostreae*** |  |  |  |  |  |
| J2-9 | - | - | - | - | [GCA_001368855.1](https://www.ncbi.nlm.nih.gov/assembly/GCA_001368855.1) |
| J5-4 | - | - | - | - | [GCA_001368875.1](https://www.ncbi.nlm.nih.gov/assembly/GCA_001368875.1) |
| 9CS106 | - | - | - | - | [GCA_000272185.2](https://www.ncbi.nlm.nih.gov/assembly/GCA_000272185.2) |
| 9ZC13 | - | - | - | - | [GCA_000272045.2](https://www.ncbi.nlm.nih.gov/assembly/GCA_000272045.2) |
| 9ZC77 | - | - | - | - | [GCA_000272065.2](https://www.ncbi.nlm.nih.gov/assembly/GCA_000272065.2) |
| 9ZC88 | - | - | - | - | [GCA_000272205.2](https://www.ncbi.nlm.nih.gov/assembly/GCA_000272205.2) |
| ZF-91 | - | - | - | - | [GCA_000272085.2](https://www.ncbi.nlm.nih.gov/assembly/GCA_000272085.2) |
| SOB6_12 | - | - | - | - | [GCA_004342375.1](https://www.ncbi.nlm.nih.gov/assembly/GCA_004342375.1) |
| SOB7_9 | - | - | - | - | [GCA_003814675.1](https://www.ncbi.nlm.nih.gov/assembly/GCA_003814675.1) |
| SOS2_11 | - | - | - | - | [GCA_004341745.1](https://www.ncbi.nlm.nih.gov/assembly/GCA_004341745.1) |
| SOS4_4 | - | - | - | - | [GCA_003814595.1](https://www.ncbi.nlm.nih.gov/assembly/GCA_003814595.1) |
| SOT2_12 | - | - | - | - | [GCA_003814775.1](https://www.ncbi.nlm.nih.gov/assembly/GCA_003814775.1) |
| SOT7_11 | - | - | - | - | [GCA_004342865.1](https://www.ncbi.nlm.nih.gov/assembly/GCA_004342865.1) |
| SOT8_11 | - | - | - | - | [GCA_003814975.1](https://www.ncbi.nlm.nih.gov/assembly/GCA_003814975.1) |
| 16BF1_28 | - | - | - | - | [GCA_004342955.1](https://www.ncbi.nlm.nih.gov/assembly/GCA_004342955.1) |
| 16BF1_56 | - | - | - | - | [GCA_004345545.1](https://www.ncbi.nlm.nih.gov/assembly/GCA_004345545.1) |
| 16BF1_95 | - | - | - | - | [GCA_004342305.1](https://www.ncbi.nlm.nih.gov/assembly/GCA_004342305.1) |
| 16BF5_48 | - | - | - | - | [GCA_004341905.1](https://www.ncbi.nlm.nih.gov/assembly/GCA_004341905.1) |
| 16SF1_51 | - | - | - | - | [GCA_003751935.1](https://www.ncbi.nlm.nih.gov/assembly/GCA_003751935.1) |
| 16SF1_87 | - | - | - | - | [GCA_003751925.1](https://www.ncbi.nlm.nih.gov/assembly/GCA_003751925.1) |
| 33_O_99 | - | - | - | - | [GCA_017917535.1](https://www.ncbi.nlm.nih.gov/assembly/GCA_017917535.1) |
| 33_O_98 | - | - | - | - | [GCA_017917555.1](https://www.ncbi.nlm.nih.gov/assembly/GCA_017917555.1) |
| 28_O_18 | - | - | - | - | [GCA_017917955.1](https://www.ncbi.nlm.nih.gov/assembly/GCA_017917955.1) |
| 28_O_21 | - | - | - | - | [GCA_017917965.1](https://www.ncbi.nlm.nih.gov/assembly/GCA_017917965.1) |
| 26_O_12 | - | - | - | - | [GCA_017918095.1](https://www.ncbi.nlm.nih.gov/assembly/GCA_017918095.1) |
| 33_O_91 | - | - | - | - | [GCA_017917635.1](https://www.ncbi.nlm.nih.gov/assembly/GCA_017917635.1) |
| 28_O_30 | - | - | - | - | [GCA_017917875.1](https://www.ncbi.nlm.nih.gov/assembly/GCA_017917875.1) |
| 7Z_4 | - | - | - | - | [GCA_017916775.1](https://www.ncbi.nlm.nih.gov/assembly/GCA_017916775.1) |
| 37_O_172 | - | - | - | - | [GCA_017917455.1](https://www.ncbi.nlm.nih.gov/assembly/GCA_017917455.1) |
| LGP15 | - | - | - | - | [GCA_017916495.1](https://www.ncbi.nlm.nih.gov/assembly/GCA_017916495.1) |
| 28_O_28 | - | - | - | - | [GCA_017917915.1](https://www.ncbi.nlm.nih.gov/assembly/GCA_017917915.1) |
| 45_O_295 | - | - | - | - | [GCA_017917195.1](https://www.ncbi.nlm.nih.gov/assembly/GCA_017917195.1) |
| 28_O_17 | - | - | - | - | [GCA_017918065.1](https://www.ncbi.nlm.nih.gov/assembly/GCA_017918065.1) |
| 42_O_252 | - | - | - | - | [GCA_017917235.1](https://www.ncbi.nlm.nih.gov/assembly/GCA_017917235.1) |
| 47_O_357 | - | - | - | - | [GCA_017916915.1](https://www.ncbi.nlm.nih.gov/assembly/GCA_017916915.1) |
| 28_O_24 | - | - | - | - | [GCA_017917995.1](https://www.ncbi.nlm.nih.gov/assembly/GCA_017917995.1) |
| J5-6 | - | - | - | - | [GCA_017916555.1](https://www.ncbi.nlm.nih.gov/assembly/GCA_017916555.1) |
| 31_O_77 | - | - | - | - | [GCA_017917735.1](https://www.ncbi.nlm.nih.gov/assembly/GCA_017917735.1) |
| 37_O_187 | - | - | - | - | [GCA_017917355.1](https://www.ncbi.nlm.nih.gov/assembly/GCA_017917355.1) |
| 7G1-1 | - | - | - | - | [GCA_018440535.1](https://www.ncbi.nlm.nih.gov/assembly/GCA_018440535.1) |
| 7D8_10 | - | - | - | - | [GCA_017916815.1](https://www.ncbi.nlm.nih.gov/assembly/GCA_017916815.1) |
| 46_O_324 | - | - | - | - | [GCA_017917055.1](https://www.ncbi.nlm.nih.gov/assembly/GCA_017917055.1) |
| 31_O_82 | - | - | - | - | [GCA_017917655.1](https://www.ncbi.nlm.nih.gov/assembly/GCA_017917655.1) |
| 7F1_18 | - | - | - | - | [GCA_017916825.1](https://www.ncbi.nlm.nih.gov/assembly/GCA_017916825.1) |
| 45_O_294 | - | - | - | - | [GCA_017917135.1](https://www.ncbi.nlm.nih.gov/assembly/GCA_017917135.1) |
| J5-23 | - | - | - | - | [GCA_017916595.1](https://www.ncbi.nlm.nih.gov/assembly/GCA_017916595.1) |
| 8F5-39 | - | - | - | - | [GCA_018440505.1](https://www.ncbi.nlm.nih.gov/assembly/GCA_018440505.1) |
| 34_O_104 | - | - | - | - | [GCA_017917495.1](https://www.ncbi.nlm.nih.gov/assembly/GCA_017917495.1) |
| 37_O_183 | - | - | - | - | [GCA_017917395.1](https://www.ncbi.nlm.nih.gov/assembly/GCA_017917395.1) |
| J2-13 | - | - | - | - | [GCA_017916615.1](https://www.ncbi.nlm.nih.gov/assembly/GCA_017916615.1) |
| 47_O_349 | - | - | - | - | [GCA_017917015.1](https://www.ncbi.nlm.nih.gov/assembly/GCA_017917015.1) |
| 31_O_72 | - | - | - | - | [GCA_017917765.1](https://www.ncbi.nlm.nih.gov/assembly/GCA_017917765.1) |
| 45_O_306 | - | - | - | - | [GCA_017918195.1](https://www.ncbi.nlm.nih.gov/assembly/GCA_017918195.1) |
| 45_O_293 | - | - | - | - | [GCA_017917215.1](https://www.ncbi.nlm.nih.gov/assembly/GCA_017917215.1) |
| 29_O_51 | - | - | - | - | [GCA_017917755.1](https://www.ncbi.nlm.nih.gov/assembly/GCA_017917755.1) |
| 45_O_291 | - | - | - | - | [GCA_017917285.1](https://www.ncbi.nlm.nih.gov/assembly/GCA_017917285.1) |
| 47_O_358 | - | - | - | - | [GCA_017916895.1](https://www.ncbi.nlm.nih.gov/assembly/GCA_017916895.1) |
| J5-24 | - | - | - | - | [GCA_017916625.1](https://www.ncbi.nlm.nih.gov/assembly/GCA_017916625.1) |
| 31_O_75 | - | - | - | - | [GCA_017917695.1](https://www.ncbi.nlm.nih.gov/assembly/GCA_017917695.1) |
| 29_O_45 | - | - | - | - | [GCA_017917815.1](https://www.ncbi.nlm.nih.gov/assembly/GCA_017917815.1) |
| 32_O_89 | - | - | - | - | [GCA_017917615.1](https://www.ncbi.nlm.nih.gov/assembly/GCA_017917615.1) |
| 38_P_218 | - | - | - | - | [GCA_017917315.1](https://www.ncbi.nlm.nih.gov/assembly/GCA_017917315.1) |
| 45_P_323 | - | - | - | - | [GCA_017917035.1](https://www.ncbi.nlm.nih.gov/assembly/GCA_017917035.1) |
| J5-28 | - | - | - | - | [GCA_017916575.1](https://www.ncbi.nlm.nih.gov/assembly/GCA_017916575.1) |
| 47_O_352 | - | - | - | - | [GCA_017916955.1](https://www.ncbi.nlm.nih.gov/assembly/GCA_017916955.1) |
| 47_O_347 | - | - | - | - | [GCA_017917075.1](https://www.ncbi.nlm.nih.gov/assembly/GCA_017917075.1) |
| 29_O_34 | - | - | - | - | [GCA_017917855.1](https://www.ncbi.nlm.nih.gov/assembly/GCA_017917855.1) |
| 46_O_330 | - | - | - | - | [GCA_017918175.1](https://www.ncbi.nlm.nih.gov/assembly/GCA_017918175.1) |
| 37_O_186 | - | - | - | - | [GCA_017917365.1](https://www.ncbi.nlm.nih.gov/assembly/GCA_017917365.1) |
| 29_O_42 | - | - | - | - | [GCA_018440585.1](https://www.ncbi.nlm.nih.gov/assembly/GCA_018440585.1) |
| 47_O_359 | - | - | - | - | [GCA_017916855.1](https://www.ncbi.nlm.nih.gov/assembly/GCA_017916855.1) |
|  |  |  |  |  |  |
